# Therapeutic targeting of metabolic vulnerabilities in cancers with MLL3/4-COMPASS epigenetic regulator mutations

**DOI:** 10.1172/JCI169993

**Published:** 2023-07-03

**Authors:** Zibo Zhao, Kaixiang Cao, Jun Watanabe, Cassandra N. Philips, Jacob M. Zeidner, Yukitomo Ishi, Qixuan Wang, Sarah R. Gold, Katherine Junkins, Elizabeth T. Bartom, Feng Yue, Navdeep S. Chandel, Rintaro Hashizume, Issam Ben-Sahra, Ali Shilatifard

**Affiliations:** 1 Department of Biochemistry and Molecular Genetics,; 2 Simpson Querrey Center for Epigenetics,; 3 Robert H. Lurie NCI Comprehensive Cancer Center, and; 4 Department of Medicine, Northwestern University Feinberg School of Medicine, Chicago, Illinois, USA.

**Keywords:** Genetics, Metabolism, Colorectal cancer, Epigenetics

## Abstract

Epigenetic status–altering mutations in chromatin-modifying enzymes are a feature of human diseases, including many cancers. However, the functional outcomes and cellular dependencies arising from these mutations remain unresolved. In this study, we investigated cellular dependencies, or vulnerabilities, that arise when enhancer function is compromised by loss of the frequently mutated COMPASS family members MLL3 and MLL4. CRISPR dropout screens in MLL3/4-depleted mouse embryonic stem cells (mESCs) revealed synthetic lethality upon suppression of purine and pyrimidine nucleotide synthesis pathways. Consistently, we observed a shift in metabolic activity toward increased purine synthesis in MLL3/4-KO mESCs. These cells also exhibited enhanced sensitivity to the purine synthesis inhibitor lometrexol, which induced a unique gene expression signature. RNA-Seq identified the top MLL3/4 target genes coinciding with suppression of purine metabolism, and tandem mass tag proteomic profiling further confirmed upregulation of purine synthesis in MLL3/4-KO cells. Mechanistically, we demonstrated that compensation by MLL1/COMPASS was underlying these effects. Finally, we demonstrated that tumors with MLL3 and/or MLL4 mutations were highly sensitive to lometrexol in vitro and in vivo, both in culture and in animal models of cancer. Our results depicted a targetable metabolic dependency arising from epigenetic factor deficiency, providing molecular insight to inform therapy for cancers with epigenetic alterations secondary to MLL3/4 COMPASS dysfunction.

## Graphical abstract

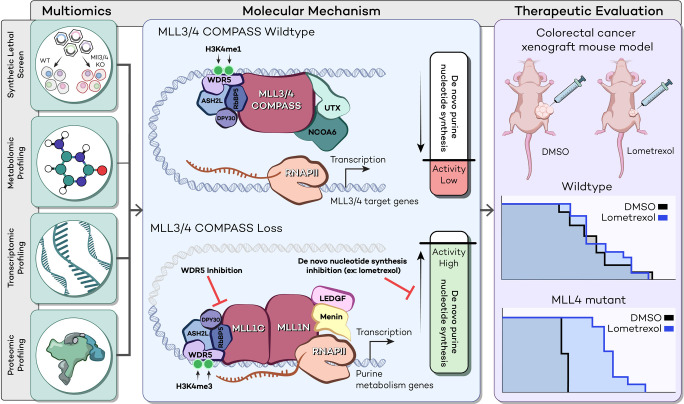

## Introduction

The COMplex of Proteins Associated with Set1 (COMPASS) family members MLL3 and MLL4 are the major H3K4 histone lysine mono-methyltransferases functioning at enhancers (1–3). Their roles in the regulation of enhancer activity and gene expression involve both the catalytic-dependent function of H3K4 mono-methylation and the catalytic-independent cofunctional association with UTX and p300 to activate enhancers (4–70). Functional activity of MLL3 and MLL4 at enhancers is essential for a variety of mammalian developmental processes including myogenesis, adipogenesis, cardiogenesis, macrophage activation, and germinal center B cell development (8). The distinct requirements for MLL3 versus MLL4 during mammalian development are stage dependent; MLL4 is required after implantation and its action is dosage dependent, while MLL3 is essential during late gestation and for organ maturation (9). In mouse embryonic stem cells (mESCs), though MLL4 is dispensable for cell identity and self renewal, it is required to exit the pluripotent state and for cell fate transition (10, 11).

The emerging role of chromatin function in normal development, as well as its dysfunction in developmental diseases and cancers, involves the orchestrated activity of chromatin-modifying enzymes, chromatin regulatory factors, and chromosomal regulatory elements. The large consortium project Catalogue Of Somatic Mutations In Cancer (COSMIC) has identified a panoply of somatic mutations affecting the chromatin-modifying enzyme factors that regulate enhancers across a wide range of human tumor types. The most frequently mutated factors include the H3K4 mono-methyltransferases MLL3 and MLL4 (encoded by the genes *KMT2C* and *KMT2D*, respectively) and their cofactor, the H3K27 demethylase UTX (*KDM6A*); the prevalence of mutations affecting these factors indicates their broad roles as tumor suppressors (12, 13). Intriguingly, mutations resulting in loss of function of MLL4 or UTX also result in developmental disorders such as Kabuki syndrome (14–16). However, despite the extensive cataloguing of these recurrent mutations in enhancer chromatin regulators across different cancer types and developmental disorders, little is known regarding the molecular mechanisms underlying their oncogenic function.

The prevalence of MLL3 and MLL4 mutations in human developmental diseases and cancers prompted us to investigate the cellular dependencies, or vulnerabilities, in MLL3/4-impaired cells. In this study, we used mESCs as a model system to identify nucleotide synthesis dependence in MLL3/4-KO cells and demonstrated synthetic lethality arising from nucleotide synthesis inhibition in MLL3/4-deficient mESCs and human cancers. Mechanistically, we show that the compensation by MLL1/COMPASS is underlying these effects. We believe that our study highlights possible treatment strategies for diseases associated with MLL3/4 loss-of-function mutations.

## Results

### Genome-wide screening identifies purine and pyrimidine synthesis pathways essential for viability in MLL3/4-KO mESCs.

To characterize the phenotypic consequences of MLL3/4 loss, we generated an MLL3/4 double–KO mESC line by deleting exons 8 and 9 of *KMT2C* (MLL3) and exons 16–22 of *KMT2D* (MLL4) (Supplemental Figure 1A; supplemental material available online with this article; https://doi.org/10.1172/JCI169993DS1). Successful knockout of MLL3 and MLL4 was confirmed by Western blot analysis using antibodies against N-terminal and mid-protein regions of MLL3, N- and C-terminal regions of MLL4, the pan-COMPASS subunit RBBP5, and the NCOA6, UTX and PTIP subunits unique to the MLL3/4 branch of COMPASS (Supplemental Figure 1B). To examine the histone modifications and gene expression pattern in MLL3/4-KO cells, we performed H3K4me1 and H3K27Ac ChIP-Seq. K-means clustering (k=2) separated the previously defined MLL4 peaks (10) into 2 groups based on the log_2_ fold change in H3K4me1 and H3K27Ac ChIP-Seq signal relative to signal in WT mESCs (Supplemental Figure 1C). Meta-analysis revealed dramatic H3K4me1 and H3K27Ac reduction coinciding with strongly reduced expression of genes at Group2 MLL4 peaks in MLL3/4-KO cells (Supplemental Figure 1C). Feature distribution of MLL4 peaks and KEGG pathway enrichment analysis of nearby genes demonstrated the similarity of MLL4 peaks in both groups. These peaks were mostly located at enhancer regions in both group 1 and group 2, as defined by K-means clustering, and were enriched in pathways related to cytoskeleton and junction organization (Supplemental Figure 1, D and E). Finally, pluripotency markers, including *Pou5f1* and *Nanog* gene expression, remained the same in WT and MLL3/4-KO cells, suggesting that MLL3/4 are dispensable for self renewal in mESCs (Supplemental Figure 1F). The cellular growth rates of WT and MLL3/4-KO cells were also comparable (Supplemental Figure 1G). Importantly, the pluripotency markers and cellular growth rates in these figures reflect the cellular status of WT and MLL3/4-KO mESCs in long-term culture conditions inherent to the generation of KO clones.

**Figure 1:**
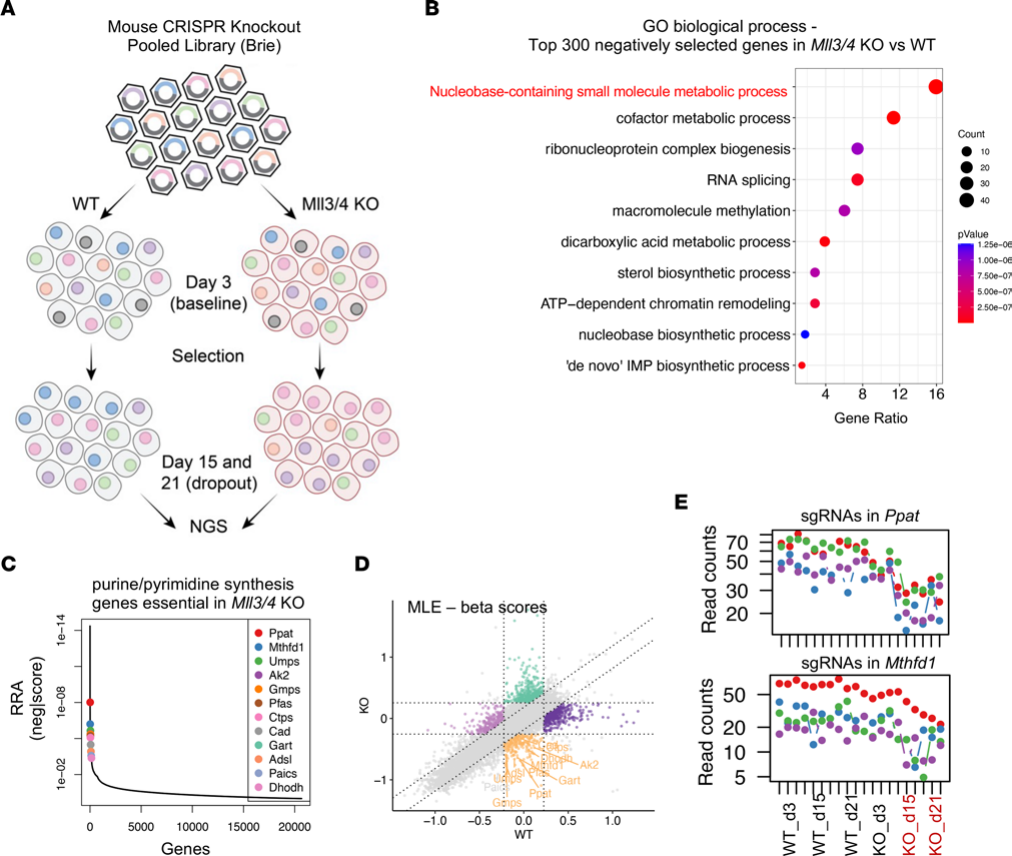
Genome-wide Screen Identifies Purine and Pyrimidine Synthesis Pathways as Essential for viability in MLL3/4*-*KO mESCs. (**A**) Flowchart showing CRISPR KO-based dropout screening in WT and KO cells. (**B**) GO biological process pathway analysis (using MAGeCK) of the top 300 negatively selected genes in MLL3/4-KO cells. (**C**) Rank plot showing the distribution of negative RRA score with the scores of the 12 genes involved in purine/pyrimidine synthesis that were identified as essential in MLL3/4 KO. (**D**) β scores of gene essentiality were calculated for WT and KO cells. The 12 screen-identified genes involved in purine/pyrimidine synthesis are shown in the 9-square view. Orange, genes selectively essential in KO not WT; light purple, genes selectively essential in WT not KO; green, genes depletion of which enhanced survival in KO not WT; dark purple, genes depletion of which enhanced survival in WT not KO. (**E**) Distribution of sgRNA read counts (normalized) of the 2 representative genes *Ppat* and *Mthfd1* in WT and KO cells at different time points. The four colors represent the four distinct sgRNAs in the Brie CRISPR library.

To investigate possible cellular vulnerabilities arising due to MLL3/4 depletion, we performed a dropout screen with the Brie CRISPR knockout library in WT and MLL3/4-KO mESCs (Figure 1A). We took day 3 as the initial baseline state and days 15 and 21 as the final state. After scrutinizing normalized read counts and sample clustering to confirm dropout versus baseline at the final state (Supplemental Figure 2A), the enrichment of sgRNAs in live cells after 15 and 21 days of selection was compared with cells harvested after 3 days of infection without selection to identify genes for which Cas9 KO caused negative selection of MLL3/4 KO relative to WT cells (Figure 1A). The top 300 negatively selected genes were enriched in pathways related to a variety of metabolic processes including nucleobase-containing small molecule metabolic process (Figure 1B). Twelve metabolic enzymes critical for purine and pyrimidine synthesis (17) were all identified as essential at the final state of our screen, as shown in the rank plot of negative RRA scores (18) (Figure 1C). To examine the MLL3/4 KO-specific essentiality of these metabolic genes, β scores of gene essentiality were calculated for WT and KO cells using the MLE algorithm (19). Indeed, all the screen-identified purine and pyrimidine de novo synthesis genes were essential in KO but not WT cells, as shown in the scatter plot of β scores (Figure 1D). This specificity was further confirmed by examining the log_2_ fold change in sgRNA enrichment for WT and MLL3/4-KO cells (Supplemental Figure 2B). For example, distribution of normalized *Ppat* and *Mthfd1* sgRNA read counts were depleted only in MLL3/4-KO cells at the final state (day 15 and day 21) (Figure 1E). Similar trends were also observed for the other screen-identified genes encoding de novo purine and pyrimidine synthesis enzymes (Supplemental Figure 2C). To validate the CRISPR screening results, WT and KO cells were labeled with the fluorescent proteins GFP and mCherry, respectively, and mixed at a 1-to-1 ratio for coculture prior to infection with individual sgRNAs. Fluorescent signals were captured with flow cytometry over the indicated time course (Supplemental Figure 2D). The normalized ratios of mCherry/GFP calculated for several negatively selected genes in MLL3/4-KO cells (including *Yrdc*, *Strap*, *Pfas*, *Zic3*, *Umps,* and *Slc25a19*) suggest that KO cells are preferentially more sensitive to the depletion of these genes by sgRNAs (Supplemental Figure 2E). Overall, our CRIPSR screen identified the existence of an emergent dependence on de novo nucleotide synthesis when the functions of MLL3 and MLL4 were compromised in mESCs.

### Loss of MLL3 and MLL4 increases flux through purine synthesis in mESCs.

Due to the emergent cellular dependence on nucleotide synthesis pathway genes we observed in MLL3/4-KO cells, we performed liquid-chromatography (LC) tandem mass spectrometry-based (MS/MS-based) steady-state metabolite profiling to globally assess the metabolome of WT and MLL3/4-KO cells (Supplemental Figure 3). Among the small molecules detected, the steady-state relative abundance of metabolites involved in methionine metabolism (S-adenosylhomocysteine, homocysteine, and cystathionine), urea cycle (arginosuccinate and carbamoyl-phosphate), and nucleotide metabolism (UMP, CMP, AICAR, carbamoyl-phosphate, inosine, adenosine, and hypoxanthine) were significantly altered in MLL3/4-deficient cells compared with WT cells (Supplemental Figure 3, C–E).

**Figure 2:**
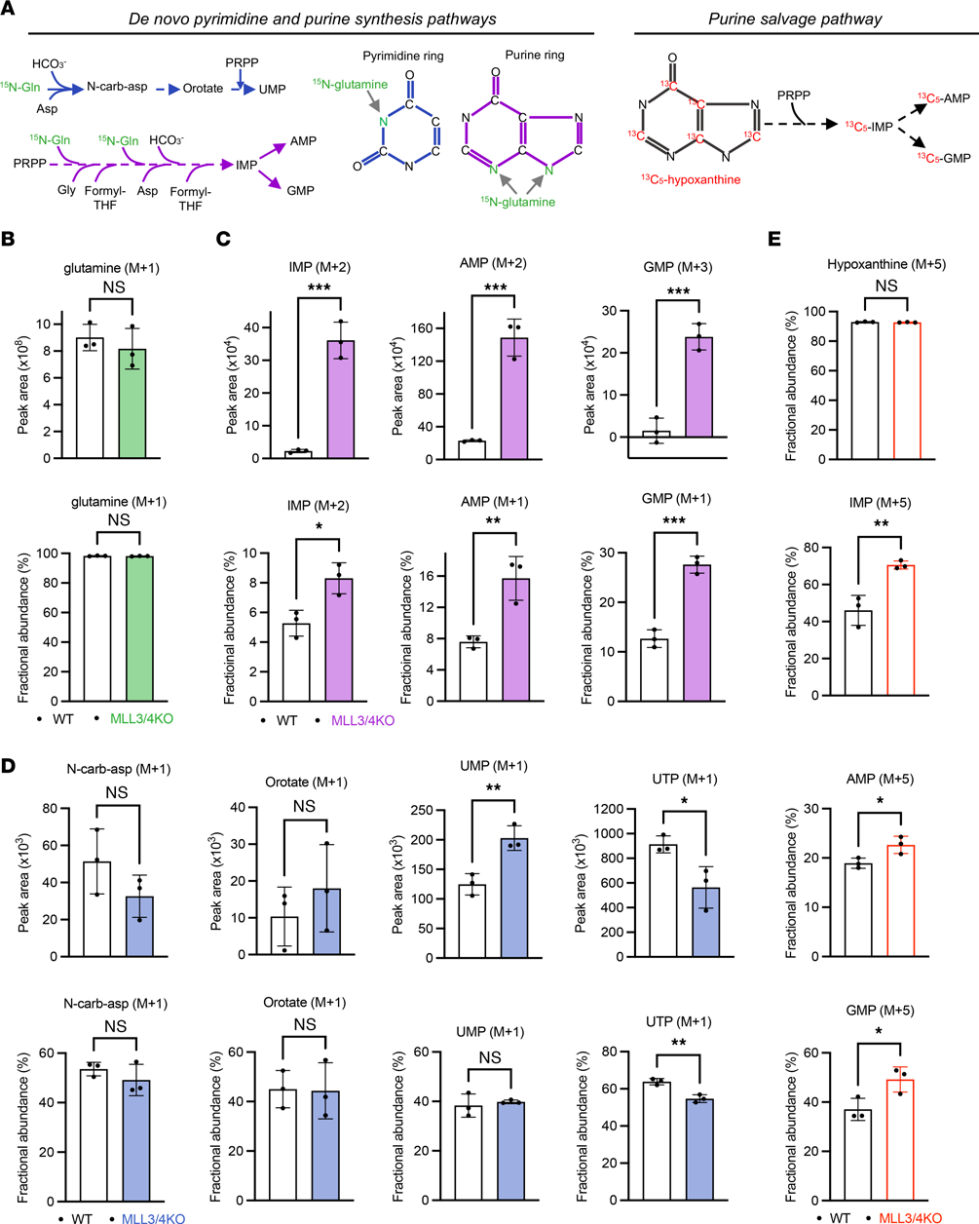
Loss of MLL3 and MLL4 increases flux through purine synthesis in mESCs. (**A**) The flowchart of ^15^N glutamine and ^13^C HTX isotope-labeled metabolite tracing. (**B**) Levels and fractional abundance (out of total glutamine) of the tracer-labeled glutamine (M+1) in WT and KO cells. (**C**) Incorporation of ^15^N tracer from labeled glutamine into purines. (**D**) Incorporation of ^15^N tracer from labeled glutamine into pyrimidines. (**E**) Incorporation of ^13^C tracer from labeled HTX into purines. WT, *n =* 3; MLL3/4 KO, *n =* 3. Data are presented as mean ± SD. *P < 0.05, **P < 0.01, ***P < 0.001 with unpaired *t* test.

Because the relative abundance of several nucleotide synthesis intermediates was altered in MLL3/4-KO cells, we decided to focus our study on the effects of MLL3/4 loss on nucleotide metabolism, a set of essential processes that maintain nucleotide pools to support nucleic acid and protein synthesis, energy transfer, signaling activity, and cytoskeletal function (17, 20). Nucleotides can be produced through the de novo synthesis or salvage pathways. The de novo synthesis pathways utilize carbon and nitrogen from specific amino acids and carbon from other small molecule donors to build the purine and pyrimidine rings. On the other hand, the purine salvage pathway recycles existing nucleosides and nucleobases, such as hypoxanthine, to maintain purine nucleotide pools. Importantly, steady-state metabolite abundance measurements alone are insufficient to determine whether the activity of a metabolic pathway is altered (21). Therefore, to further investigate whether the increased nucleotide levels observed in the global profile of MLL3/4-KO cells reflect increased flux through either the de novo or purine salvage pathways (Figure 2A), we employed LC-MS/MS-based heavy isotope-labeled metabolite tracing, including ^15^N-(amide)-glutamine and ^13^C-hypoxanthine. The ^15^N-glutamine tracing study revealed that MLL3/4-KO cells exhibit significantly increased flux through de novo purine but not pyrimidine synthesis (Figure 2, B–D). Interestingly, the ^13^C-hypoxanthine tracing study also showed that activity of the purine salvage pathway is also slightly increased in MLL3/4-KO cells (Figure 2E). Overall, these metabolic studies suggest that cells depleted of MLL3 and MLL4 have a higher flux through purine synthesis, consistent with greater dependence on purine metabolic pathway gene expression for survival.

### MLL3/4-KO mESCs display enhanced sensitivity to purine synthesis inhibition.

**Figure 3:**
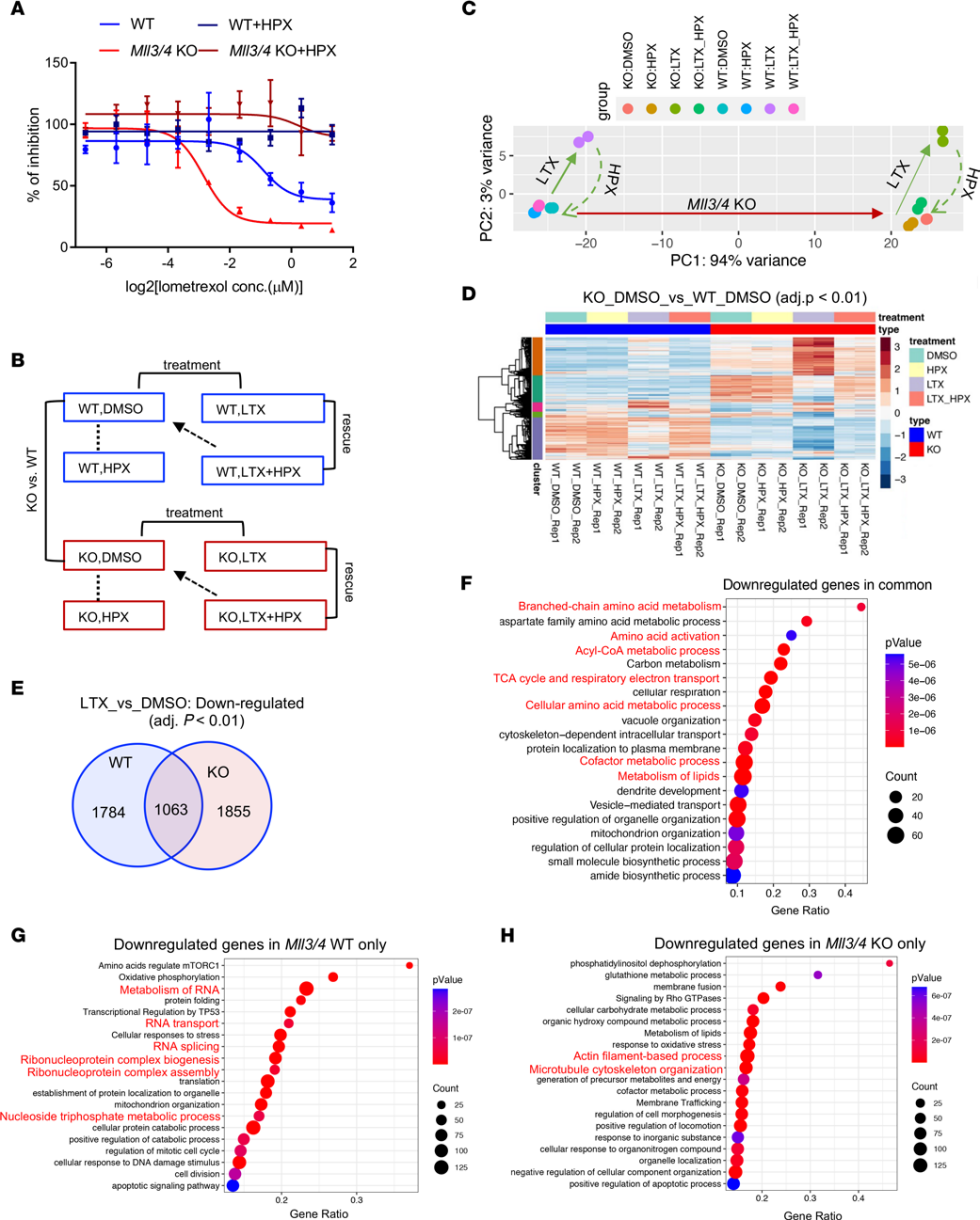
MLL3/4-KO mESCs display enhanced sensitivity to purine synthesis inhibition. (**A**) WT and MLL3/4-KO cells were treated with 0–2.5 μM LTX with a 2-fold dilution in the presence or absence of 50 μM HPX for 48 hours. A CellTiter-Glo luminescent cell viability assay was performed to determine the percentage of cell growth inhibition under these conditions. WT, *n =* 3; MLL3/4 KO, *n =* 3. (**B**) WT and KO cells were treated with 0.3 μM LTX ± 50 μM HPX for 48 hours, and cells were harvested for RNA-Seq (*n =* 2 for each condition). Differential gene expression analyses were performed with the indicated comparisons. (**C**) PC analysis of RNA-Seq in **B**. The 500 most variable genes among all the conditions were included by default. (**D**) Hierarchical clustering heatmap showing differentially expressed genes in KO cells compared with WT cells. Differentially expressed genes with Benjamini-Hochburg *P*_adj_ *<* 0.01 were selected. (**E**) Venn diagram showing the overlap of genes downregulated upon LTX treatment in WT or KO cells. (**F**) Pathway enrichment analysis of the genes downregulated upon LTX treatment in both WT and KO cells. (**G**) Pathway enrichment analysis of the genes uniquely downregulated upon LTX treatment in WT cells. (**H**) Pathway enrichment analysis of the genes uniquely downregulated upon LTX treatment in MLL3/4-KO cells.

We hypothesized that MLL3/4-KO cells would have enhanced sensitivity to purine synthesis inhibition due to their increased use of and dependence on purine nucleotide synthesis pathways. WT and MLL3/4-KO cells were treated with lometrexol (LTX), a de novo purine synthesis inhibitor that specifically targets glycinamide ribonucleotide formyltransferase (GART). The purine salvage pathway nucleobase hypoxanthine (HPX) was added to bypass the LTX-inhibited de novo purine synthesis pathway (Figure 3A). We observed elevated sensitivity to LTX in MLL3/4-KO cells, with increased inhibition of cell growth and/or viability relative to WT cells, which was completely rescued by HPX (Figure 3A and Supplemental Figure 4A). If MLL3/4-KO cells gain sensitivity to de novo purine synthesis inhibition, we hypothesize that they should also be sensitive to inhibition of pathways upstream of purine synthesis, including folate-mediated 1-carbon (1C) metabolism. Indeed, MLL3/4-KO cells showed enhanced sensitivity to the inhibitors methotrexate (MTX) and SHIN1 (Supplemental Figure 4, B and C). MTX targets dihydrofolate reductase (DHFR), inhibiting tetrahydrofolate (THF) regeneration, and SHIN1 targets serine hydroxymethyltransferase 1 and serine hydroxymethyltransferase 2 (SHMT1/2), inhibiting the transfer of a carbon from serine onto THF to produce 5,10 methylene-THF; both inhibitors restrict the availability of the 1C donors required for de novo purine synthesis. To elucidate the mechanism of differential sensitivity to LTX treatment in the presence or absence of MLL3/4, we first performed RNA-Seq to compare the changes in gene expression in WT and KO cells in response to LTX (0.3 μM) and/or HPX treatment (Figure 3B). The LTX concentration chosen was close to the IC_50_ determined in MLL3/4-KO cells. Interestingly, principal component (PC) analysis demonstrated that the vast majority of variance in gene expression was explained by PC1 (94%), which effectively separated the samples by genotype (Figure 3C). HPX alone elicited subtle gene expression changes, whereas LTX treatment induced significant gene expression changes, which were also almost completely rescued by HPX in both WT and MLL3/4-KO cells (Figure 3C). The expression of genes differentially expressed between WT and KO cells was further altered in response to LTX treatment, with a more prominent effect in KO cells; a significant number of the genes upregulated in KO cells were further elevated by LTX treatment (top cluster in the KO_LTX group), while genes downregulated in KO cells were further depressed (bottom cluster in the KO_LTX group) (Figure 3D). The differential sensitivity to LTX treatment prompted us to investigate the gene expression changes shared between or unique to either WT or KO cells. Beginning with downregulated genes, we found 1,063 genes downregulated upon LTX treatment in both WT and KO cells (Figure 3E). Pathway enrichment analysis demonstrated that these in-common downregulated genes were enriched for biological processes related to amino acid and cofactor metabolism, which was an expected outcome of GART targeting and purine synthesis inhibition (Figure 3F). When analyzing the pathway enrichment of genes uniquely downregulated in WT cells upon LTX treatment, we identified processes related to RNA and its metabolism (Figure 3G). In contrast, the genes uniquely downregulated in MLL3/4-KO cells upon LTX treatment were enriched in pathways related to actin filament-based process and microtubule cytoskeleton organization (Figure 3H). Examining upregulated genes, we found 1,195 genes upregulated upon LTX treatment in both WT and MLL3/4-KO cells(Supplemental Figure 4D), which were enriched for a variety of biological processes including chromatin organization, cellular response to DNA damage, and stress stimulus (Supplemental Figure 4E). Interestingly, several of the enriched pathways for the genes uniquely upregulated in either WT or KO cells were also reciprocally enriched for the uniquely downregulated genes in the opposite genotype (Supplemental Figure 4, F and G; reciprocal pathways labeled in red). The distinct gene expression signature changes in the presence or absence of MLL3/4 could potentially predict the differential sensitivity to LTX treatment.

To investigate whether the differential purine biosynthesis inhibition sensitivity in the presence or absence of MLL3/4 is due to the loss of catalytic or catalysis-independent activity, we compared the effects of LTX treatment in WT, MLL3/4 ΔSET (22), and MLL3/4-KO cells. Loss of SET domains did not affect the stability of COMPASS subunits specific to MLL3/4, including NCOA6 and UTX, and bulk H3K4me1 and H3K4me2 levels were reduced to a similar degree in both MLL3/4 ΔSET and MLL3/4-KO cells when compared with WT levels (Supplemental Figure 5A). Of note, a shift of UTX proteins from the nucleus to the cytosol and downregulation of NCOA6 were only observed in complete KO but not MLL3/4 ΔSET cells (Supplemental Figure 5B). Finally, we showed that the sensitivity to LTX in MLL3/4-KO cells is largely, if not entirely, due to catalysis-independent activities of MLL3/4, as the LTX sensitivity exhibited by MLL3/4 ΔSET was indistinguishable from that of WT mESCs (Supplemental Figure 5C).

### Tandem mass tag proteomics profiling identifies purine metabolism upregulation in MLL3/4-KO cells.

**Figure 4:**
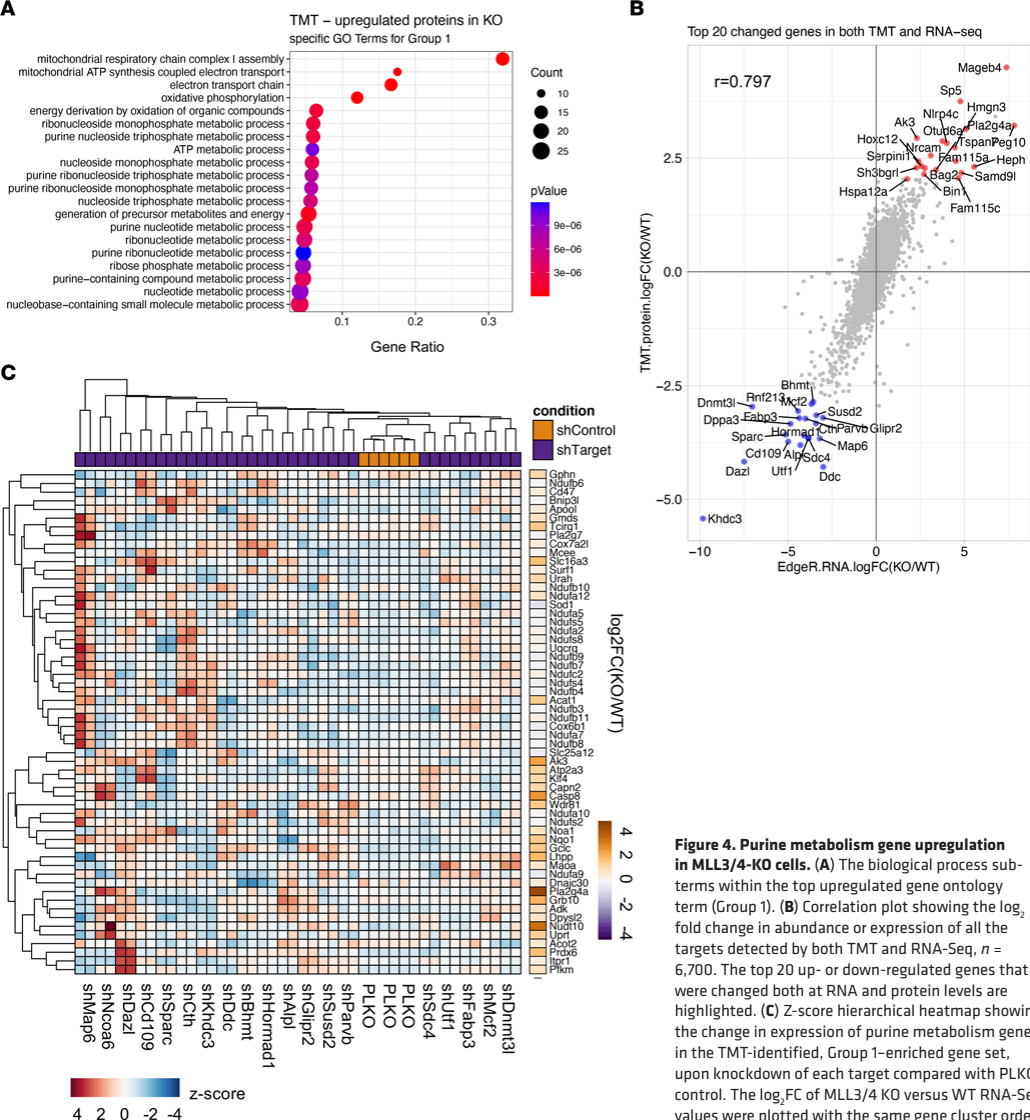
Purine metabolism gene upregulation in MLL3/4-KO cells. (**A**) The biological process sub-terms within the top upregulated gene ontology term (Group 1). (**B**) Correlation plot showing the log_2_ fold change in abundance or expression of all the targets detected by both TMT and RNA-Seq, *n* = 6,700. The top 20 up- or down-regulated genes that were changed both at RNA and protein levels are highlighted. (**C**) Z-score hierarchical heatmap showing the change in expression of purine metabolism genes in the TMT-identified, Group 1–enriched gene set, upon knockdown of each target compared with PLKO control. The log_2_FC of MLL3/4 KO versus WT RNA-Seq values were plotted with the same gene cluster order.

Due to the profound change in gene expression in WT and KO cells (~ 4,500 down- or upregulated genes with *P*_adj_ *<* 0.01), it is difficult to dissect which of these significant gene/protein signature changes lead to the metabolic dependency shift and other cellular defects seen in MLL3/4-KO cells. Therefore, as a complement to the RNA-Seq approach that we used to identify transcriptomic changes, we also used a tandem mass tag (TMT) approach to identify proteomic changes between WT and MLL3/4-KO cells. In summary, we identified 7,379 total collapsed proteins, 7,096 quantified proteins, and 57,479 quantified peptides. PC analysis demonstrated the separation of genotype by PC1 using all 7,096 proteins identified in TMT (Supplemental Figure 6A). A stringent cutoff (fold change > 1.75, *P* < 0.01) identified 343 proteins upregulated and 384 proteins downregulated in MLL3/4-KO cells (Supplemental Figure 6B). We further confirmed that the protein levels of MLL3/4 COMPASS components identified and quantified by our TMT approach were consistent with our previous Western blot analysis, with significantly diminished levels of MLL4 (*Kmt2d*), NCOA6, UTX (*Kdm6a*), and PTIP (*Paxip1*) (Supplemental Figure 1B and Supplemental Figure 6C). Pathway enrichment analysis of the proteins downregulated in KO cells yielded several processes related to cytoskeleton organization, cell shape regulation, cell junction organization, and cell-cell adhesion (Supplemental Figure 6D), terms consistent with the KEGG pathway enrichment analysis of genes at annotated MLL4 peaks (Supplemental Figure 1E). Among the biological processes enriched for proteins upregulated in MLL3/4-KO cells, there was significant enrichment of the mitochondrial respiratory chain complex I pathway group (Supplemental Figure 6E). The sub-terms within this group (group 1) were all related to nucleotide metabolic pathways (Figure 4A), in accordance with the increased de novo purine synthesis and purine salvage activity in MLL3/4-KO cells (Figure 2, C and E). It is important to note that a major function of the mitochondrial electron transport chain, which is also enriched, is to support nucleotide metabolism (23). Further, we identified 59 proteins related to the purine nucleotide metabolic processes in group 1 that are differentially abundant between WT and KO cells (Supplemental Figure 6F), suggesting that MLL3/4-KO cells also exhibit elevated expression of purine metabolism genes at the protein level. Collectively, we observed that loss of MLL3/4 functions rewired cells to require higher demand for purine and/or pyrimidine nucleotide synthesis for viability and survival.

### Mechanisms of purine metabolism upregulation in MLL3/4-KO cells.

Considering the possibility that MLL3/4 could indirectly downregulate the expression of nucleotide synthesis genes by directly targeting and upregulating genes encoding secondary effectors, we integrated the results of our RNA-Seq transcriptomics and TMT proteomics studies to seek top MLL3/4 target gene candidates. RNA and protein changes were consistent overall, with a correlation of 0.797 (Figure 4B). Our correlation analysis also identified factors with changes at the protein but not RNA level, including MLL4 and NCOA6 (Supplemental Figure 7A). The top 20 target effector candidates with downregulation at both the RNA and protein levels were selected for further functional analyses (Figure 4B). We found a significant reduction of H3K4me1 and H3K27Ac and an increase of H3K27me3 surrounding the enhancers of these genes, consistent with direct targets of MLL3/4-mediated transcriptional regulation (Supplemental Figure 7, B–E). Next, we knocked down each of the target effector candidate genes with shRNAs and analyzed the RNA-Seq data after batch effect removal (except for *Rnf213* and *Dppa3*, due to the unavailability of efficient shRNAs) (Supplemental Figure 7F). Ncoa6 was also included as a control, because it was the most downregulated COMPASS subunit at the protein — but not RNA — level when MLL3 and/or 4 were depleted (Supplemental Figure 1B, Supplemental Figure 6C, and Supplemental Figure 7A). First, we confirmed the knockdown efficiency of the individual shRNAs (Supplemental Figure 7F). Interestingly, *Ncoa6* knockdown also led to the downregulation of most of these top MLL3/4 targets (Supplemental Figure 7F). A significant overlap of gene regulation was found comparing *Ncoa6* knockdown with MLL3/4 depletion, consistent with the cofunctional association of MLL3/4 and NCOA6 in COMPASS (Supplemental Figure 7G). Then, we analyzed the effect of target candidate shRNA knockdown on the expression of purine metabolism genes within the TMT-identified and Group 1-enriched set, for which protein expression was found to be elevated after MLL3/4 depletion (Supplemental Figure 6F). Individual knockdown of any of several target effector candidate genes, including *Map6*, *Khdc3*, *Glipr2*, *Mcf2*, *Susd2*, and *Ncoa6*, yielded partial upregulation of the purine metabolism gene set (Figure 4C). A number of these putative MLL3/4 target effectors are involved in spindle and cytoskeletal function; whether altered expression of these genes might induce changes in cell shape or state and thereby impart the metabolic dependency seen in the MLL3/4-deficient condition may warrant further investigation.

**Figure 5:**
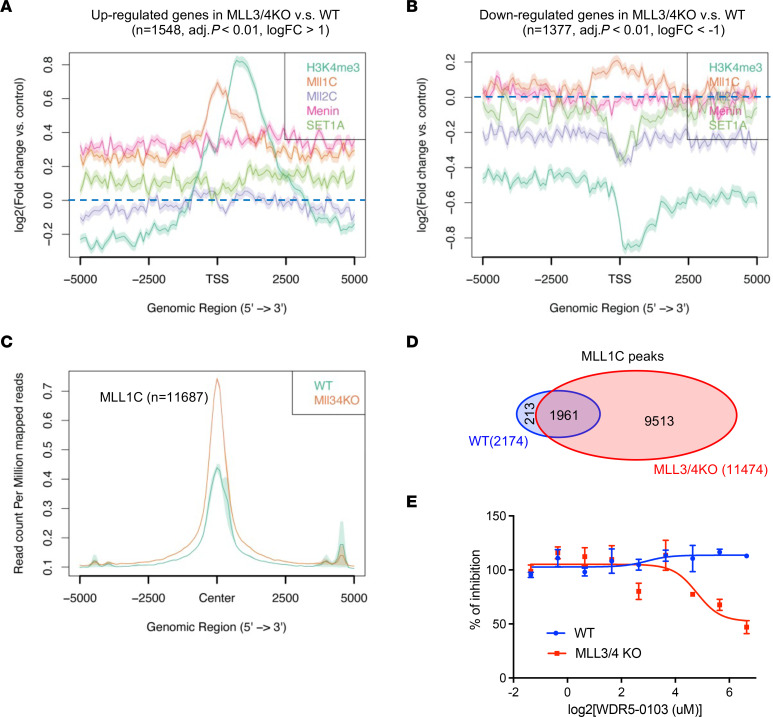
Gene expression compensation by MLL1/COMPASS in MLL3/4-KO cells. (**A**) MLL3/4 KO versus WT log_2_FC average plot showing the relative genomic binding of H3K4me3, MLL1, MLL2, Menin, and SET1A surrounding the TSS of genes that were significantly upregulated in MLL3/4-KO versus WT cells (selected based on *P*_adj_ *<* 0.01 and logFC > 1, *n =* 1548). (**B**) The significantly downregulated genes in MLL3/4 KO v.s. WT were selected based on *P*_adj_ *<* 0.01 and logFC < –1. The log_2_FC average plot showing the relative genomic binding of H3K4me3, MLL1, MLL2, Menin, and SET1A surrounding the TSS of genes that were significantly downregulated in MLL3/4 KO versus WT (selected based on *P*_adj_ *<* 0.01 and logFC < –1, *n =* 1,377). (**C**) MLL1C (MLL1 Carboxyl-terminal antibody) ChIP-Seq peaks in both WT and MLL3/4-KO cells were merged and sorted. Average plot showing the MLL1C signal in WT and MLL3/4-KO cells centered on all MLL1 peaks. *n =* 11,687. (**D**) Venn diagram showing the overlap of MLL1C peaks found in WT and MLL3/4-KO cells. Approximately 82.9% of MLL1C peaks detected in MLL3/4-KO cells are de novo peaks. (**E**) Change in cell viability of WT and MLL3/4-KO cells in response to WDR5-0103 inhibitor treatment. *n =* 3 for each specific concentration in WT and MLL3/4 KO.

To further address how the expression of purine metabolism genes is upregulated in the MLL3/4-deficient condition, we used bioinformatic tools including binding analysis for regulation of transcription (BART) (24) and epigenetic landscape in silico deletion analysis (LISA) (25) to predict the upstream transcriptional and epigenetic regulators of the 59 purine metabolism genes shown in Figure 4C. Interestingly, multiple subunits within COMPASS (WDR5, ASH2L, MLL2, etc.) were predicted as top candidate regulators of these genes. Based on this analysis, we hypothesized that the loss of MLL3/4 may cause a redistribution of the WDR5-RbBP5-ASH2L-DPY30 (WRAD) complex subunits within COMPASS and compensation from other COMPASS methyltransferases. To address this, we first performed ChIP-Seq for H3K4me3, MLL1, MLL2, Menin, and SET1A in WT and MLL3/4-KO cells. SET1B was not assessed due to its primarily cytoplasmic localization (26). Centered on the TSS regions of the genes significantly upregulated in MLL3/4-KO versus WT cells, increased H3K4me3 was accompanied by a remarkable increase in MLL1 and Menin occupancy (Figure 5A). Centered on the TSS regions of the genes significantly downregulated in MLL3/4-KO versus WT cells, decreased H3K4me3 was accompanied by a decrease in the occupancy of both MLL2 and SET1A (Figure 5B). These findings triggered us to further investigate the effect of MLL3/4 KO on the global chromatin occupancy of each of the COMPASS members. Indeed, MLL1 chromatin binding was increased in MLL3/4-KO cells (Figure 5C), while total levels of H3K4me3, MLL2, and SET1A remained the same or were slightly reduced (Supplemental Figure 8, A–C). Importantly, MLL1 chromatin binding was increased at over 80% of the purine metabolism genes that were upregulated in MLL3/4 KO(as identified by integrated RNA-Seq and TMT analysis). In addition, approximately 82.9% of MLL1C peaks detected in MLL3/4-KO cells were de novo peaks (Figure 5D), suggesting a potential gain of MLL1/COMPASS activity in cells lacking MLL3/4. Then, using inhibitors to selectively target interactions of MLL1 and/or MLL2 with other COMPASS subunits, we found that selective inhibition of the WDR5-MLL1 — but not Menin-MLL1/2 — interactions could decrease the viability of MLL3/4-KO cells (Figure 5E and Supplemental Figure 8, D–F).Selective WDR5-MLL1 inhibition decreased MLL3/4 KO cell viability via gene-specific effects. For instance, increased occupancy of H3K4me3 and MLL1 were observed at the *Nqo1* gene locus in MLL3/4-KO versus WT cells, while other marks remained the same or were diminished (Supplemental Figure 8G). *Nqo1* and its fellow purine metabolism genes, including *Ak3,* were also downregulated upon WDR5-0103 treatment in MLL3/4-KO cells (Supplemental Figure 8, H and I), suggesting that the upregulation of purine metabolism in MLL3/4-KO cells can be attributed, at least in part, to compensation specifically by MLL1/COMPASS.

We also considered the intriguing possibility that metabolic reprogramming in MLL3/4-deficient cells might be the consequence of a higher order chromatin structural change due to MLL3/4 loss. To investigate this potential mechanism, we performed Hi-C in WT and MLL3/4-KO mESCs to explore the relationship between epigenetic alterations and chromatin. The loop number in MLL3/4 KO, at 13,695, was higher than in WT, which was 10,118, and MLL3/4 KO had more open chromatin (larger ratio of A:B compartmentalization) than WT cells (Supplemental Figure 9A). To quantitate and visualize compartment strength, we plotted interaction frequencies along the first eigenvector to generate compartmentalization saddle plots (Supplemental Figure 9, B and C). The results revealed stronger A-A and weaker B-B interactions in MLL3/4-KO relative to WT cells (Supplemental Figure 9, B and C). The majority of A and B genomic compartment occupancy is unaltered upon MLL3/4 depletion, but regions with differential compartmentalization primarily undergo decompaction in MLL3/4-KO versus WT cells or shift from the more closed B to the more open A chromatin compartment; 6.79% of bins shifted from B to A (B-A) and 2.01% from A to B (A-B) (Supplemental Figure 9D). To correlate differential gene expression with differential A/B compartmentalization, we examined the differential expression of genes located within the B-A or A-B bins. A significant upregulation in MLL3/4-KO cells relative to WT was observed for genes located within B-A bins, whereas gene expression was largely unaffected for unchanged or A-B bins (Supplemental Figure 9E). Consistently, the eigenvector values at the TSS of the top upregulated genes in MLL3/4-KO cells were significantly higher than those in WT cells (Supplemental Figure 9F). In contrast, the eigenvector values at the TSS of the top downregulated genes in MLL3/4-KO cells were indistinguishable from those in WT cells (Supplemental Figure 9G). In summary, MLL3/4 loss changes higher-order chromatin structure, adding another layer of gene expression alteration that may potentially contribute to the emergent metabolic dependency.

### MLL4-mutant colorectal cancer cells are selectively sensitive to LTX treatment.

MLL4 is highly mutated in a variety of hematological malignancies and solid tumors. Some of the loss-of-function mutations are believed to function as driver mutations, conferring competitive advantages for clonal expansion (27). Given our studies in mESCs (Figures 1–5), we sought to examine whether cancer cells bearing MLL4 loss-of-function mutations share similarly altered gene expression profiles. We focused on colorectal cancer, which has a relatively high rate of MLL4 mutation, and we began by examining CCLE colorectal cancer cell line RNA-Seq data. When we analyzed the contribution of all cell line genotypes to the PCs of the combined data, we observed a tendency of truncation mutation status to segregate the different cell lines along PC2, suggesting that genes anticorrelated with PC2 might be dysregulated as a result of MLL4 truncation mutation (Supplemental Figure 10A). The top genes anticorrelated with PC2 (genes upregulated in MLL4 truncation versus WT cell lines, including BCL2, PIAS2, and E2F2) were enriched in intrinsic apoptotic signaling by p53 class mediator, cell projection morphogenesis, and protein polyubiquitination pathway terms (Supplemental Figure 10B). On the other hand, the top genes correlated with PC2 (genes downregulated in MLL4 truncation versus WT cell lines, including MMP7, APOE, and ACE2) were enriched in matrisome associated, myeloid leukocyte activation, response to wounding, inflammatory response, extracellular matrix organization, and hemostasis pathway terms (Supplemental Figure 10C). Similar GO terms were also enriched for genes downregulated in MLL3/4 KO versus WT mESCs (e.g., cytoskeleton organization, matrisome associated, and cell junction organization) (Supplemental Figure 1E and Supplemental Figure 6D), suggesting that loss-of-function MLL4 mutation in colorectal cancer shares similar gene expression features with mESC depleted of MLL3/4. We further examined the differentially expressed genes in MLL4 truncation and WT patient samples from TCGA PanCancer Atlas (Supplemental Figure 10, D and E). Some of the key pathways enriched for the genes downregulated in MLL4 truncation versus WT were consistent between colorectal cancer cell lines and patient samples, including cell junction organization and matrisome associated pathways (Supplemental Figure 10, D and F). One of the crucial consequences of MLL4 truncation mutation found in patient samples is the upregulation of immune response activation (Supplemental Figure 10E), which was missing from in vitro cell line studies. This finding is consistent with a previous study showing MLL4 deficiency sensitizes tumors to immune checkpoint blockade (28), and that MLL4 mutation status (especially truncation mutations) in cancer patients may predict response to immunotherapy and influence patient stratification and decision making.

**Figure 6:**
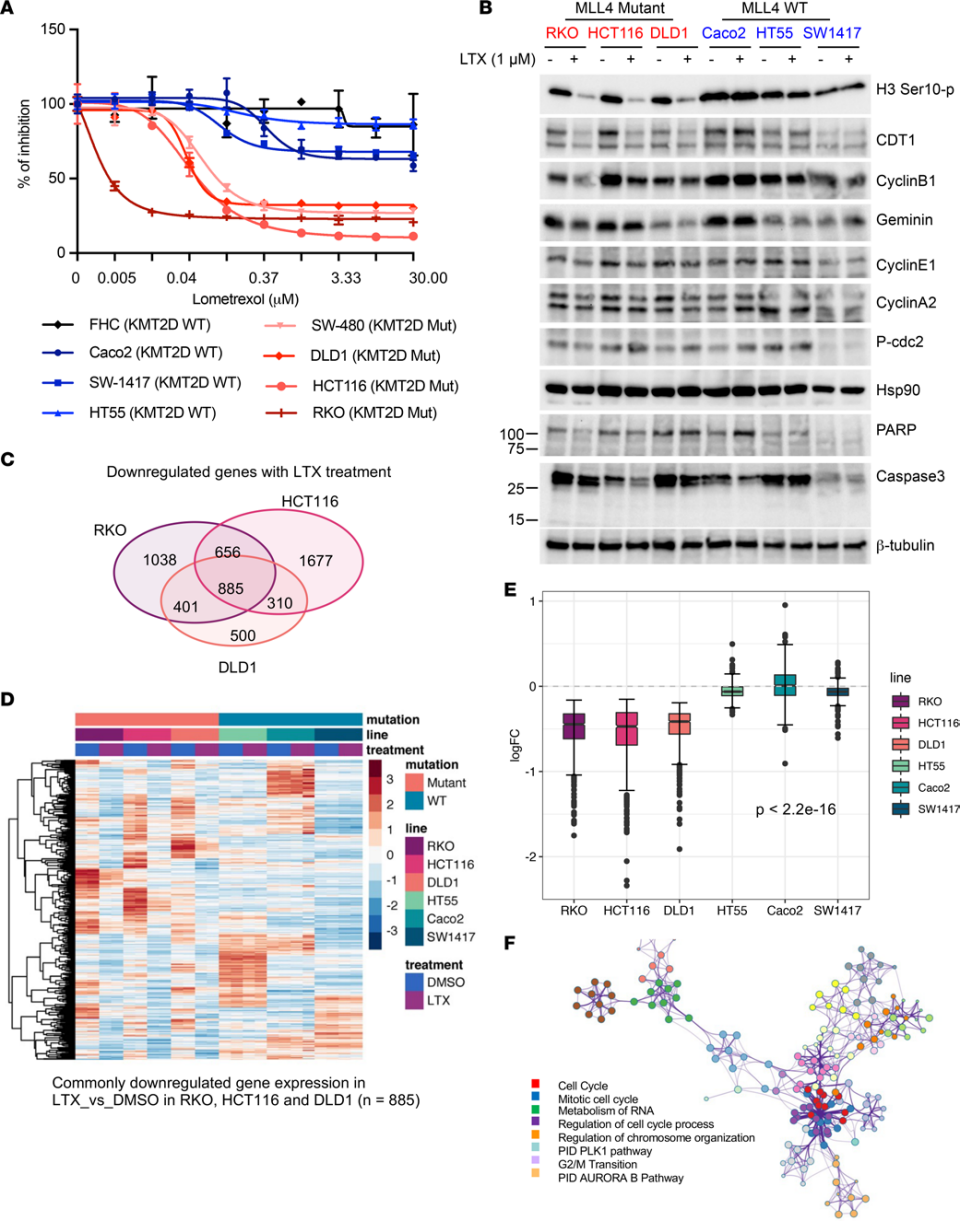
MLL4 mutant colorectal cancer cells are selectively sensitive to LTX treatment. (**A**) Cells were treated with 0–30 μM LTX with 3-fold dilution for 72 hours. A CellTiter-Glo luminescent cell viability assay was performed to determine the percentage of inhibition under these conditions. The line plot compares the sensitivity of all cell lines tested. *n =* 3 for each specific concentration in each cell line. (**B**) Cells were treated with DMSO or 1 μM LTX for 24 hours and whole cell lysates were used for Western blot against cell cycle (H3 Ser10-p, CDT1, CyclinB1, Geminin, CyclinE1, CyclinA2, and p-cdc2) and apoptosis (PARP and Caspase3) markers using Hsp90 and β-tubulin as loading controls. (**C**) Venn diagram showing the overlap of genes downregulated by LTX treatment versus control in RKO, HCT116, and DLD1 cells. (**D**) Hierarchical clustering heatmap showing the genes with expression downregulated by LTX treatment versus control in common among RKO, HCT116, and DLD1 cells (*n =* 885). (**E**) Box plot showing the logFC of the 885 downregulated genes in common among RKO, HCT116, and DLD1 cells upon LTX treatment. An unpaired *t* test was used to calculate the *P* value. Gene expression was significantly different in all the MLL4 mutant cell lines compared with MLL4 WT cells. (**F**) A network was constructed from a subset of representative cluster terms. Each term is represented by a circular node, where node size is proportional to the number of input genes falling under that term, and where color represents cluster identity. Terms with a similarity score > 0.3 are linked by an edge (for which thickness represents the similarity score). The network was visualized using Cytoscape with “force-directed” layout and with edges bundled for clarity. One representative term from each cluster was selected to be labeled with its term description.

Next, we sought to examine whether colorectal cancer cells bearing MLL4 mutations exhibited elevated sensitivity to LTX treatment. Along with 8 colorectal cancer cell lines were selected for comparison, including MLL4 WT (Caco2, SW1417, and HT55) and MLL4 mutant (SW480, DLD1, HCT116, and RKO) cell lines (Supplemental Figure 11A). It is worth noting that all of these cancer cell lines also have MLL3 but not UTX mutations (Supplemental Figure 11A), which recapitulate the low UTX mutation rate in colorectal cancer patients. Our studies showed that cell lines harboring MLL4 mutations, especially truncation mutations, were selectively more sensitive to LTX treatment than their MLL4 WT counterparts (Figure 6A and Supplemental Figure 11, B and C), consistent with our results in WT versus MLL3/4-KO mESCs and indicating that purine nucleotide synthesis may be a general synthetic lethal pathway in MLL3/4 COMPASS–deficient cells. Due to the impact of MLL4 on mitochondrial metabolism apparent in the upregulation of genes enriched in the mitochondrial respiratory chain complex I pathway in MLL3/4-KO mESCs (Supplemental Figure 6E), we separately treated colorectal cancer cells with the mitochondrial Complex I inhibitors Piericidin A or Phenformin. However, we observed no difference in sensitivity to these Complex I inhibitors between MLL4 WT and mutant cells (Supplemental Figure 11, D and E).

We further employed ^15^N-glutamine tracing in MLL4 WT (SW1417 and Caco2) and MLL4 truncation mutation (RKO and HCT116) colorectal cancer cells and demonstrated that mutant cells exhibited increased flux through de novo purine synthesis relative to their WT counterparts (Supplemental Figure 12, A and B). Next, we generated MLL4 truncation mutated (MLL4hNTD) or MLL4 complete knockout (MLL4 KO) CAL51 breast cancer cells and measured sensitivity to MTX. CAL51 cells with compromised MLL4 functions gain elevated MTX sensitivity (Supplemental Figure 12C). The cytotoxic effect of MTX on MLL4 KO cells was rescued by inosine but not thymidine, indicating that inhibition of folate regeneration by MTX exerts cytotoxic effects in the MLL4-compromised condition via secondary inhibition of purine but not dTMP synthesis (Supplemental Figure 12D). We also knocked down GART and PAICS, 2 key enzymes for de novo purine synthesis, in colorectal cancer cells (Supplemental Figure 12E). Both cellular growth and colony formation were significantly decreased by shRNA depletion of GART or PAICS in MLL4 mutant (RKO and HCT116) but not MLL4 WT (Caco2 and HT55) cell lines (Supplemental Figure 12, F–J). Our data demonstrate that cancer cells with compromised MLL4 function have elevated flux through purine synthesis and are more sensitive to inhibitors that specifically target de novo purine synthesis.

### LTX elicited downregulated expression of mitotic cell cycle genes in MLL4-mutant cancer cells.

To investigate the potential mechanisms by which LTX induced differentially cytotoxic effects in MLL4 WT and mutant cells, we examined gross cellular morphology and a panel of cell cycle and apoptosis markers (Supplemental Figure 13A and Figure 6B). In addition to mutant-specific morphological changes, a significant downregulation of histone H3 Serine10 phosphorylation (H3 Ser10-p), CDT1 and CyclinB1 was observed in MLL4 mutant but not WT cells, indicating that LTX induced cell cycle arrest and mitotic defects (Figure 6B). We further examined the gene expression change 24 hours after LTX treatment to define a unique gene expression signature associated with LTX. Consistent with the induced morphological change and cell cycle defect, LTX only induced a significant differential gene expression change in MLL4 mutant cells (Supplemental Figure 13B). In total, there were 885 genes downregulated by LTX treatment in common among all MLL4 mutant cell lines (Figure 6, C–E), and these genes were also unaffected in MLL4 WT cells (Figure 6, D and E). A total of 707 genes were upregulated by LTX treatment in common among all MLL4 mutant cell lines that were also unaffected in MLL4 WT cells (Supplemental Figure 13, C–E). LTX-downregulated genes were involved in mitotic cell cycle, regulation of chromosome organization, PLK and AURORA kinase pathways, and G2/M transition (Figure 6F). For example, expression of the genes PLK1 and AURKA was remarkably diminished in all MLL4 mutant cell lines in response to LTX, while their expression was unchanged in MLL4 WT cells (Supplemental Figure 14, A and B). Out of the 885 genes downregulated in common among MLL4 mutant cell lines, we focused on a collection of 217 genes related to mitotic cell cycle pathways and examined their gene expression in MLL4 mutant cells. The top 20 downregulated genes involved in mitotic cell cycle pathways in each cell line were identified in volcano plots (Supplemental Figure 14C). From these, we next defined a LTX-responsive mitotic gene signature, including PLK1, AURKA, CDCA3, CDC20, SFPQ, POLA1, PSRC1, KIF20A, FAM83D, and DLGAP5, the top ten downregulated genes in each MLL4 mutant cell line, for which LTX treatment had no effect in MLL4 WT cells (Supplemental Figure 14D).

### Inhibition of de novo purine synthesis by LTX inhibits MLL4 mutant tumor growth.

**Figure 7:**
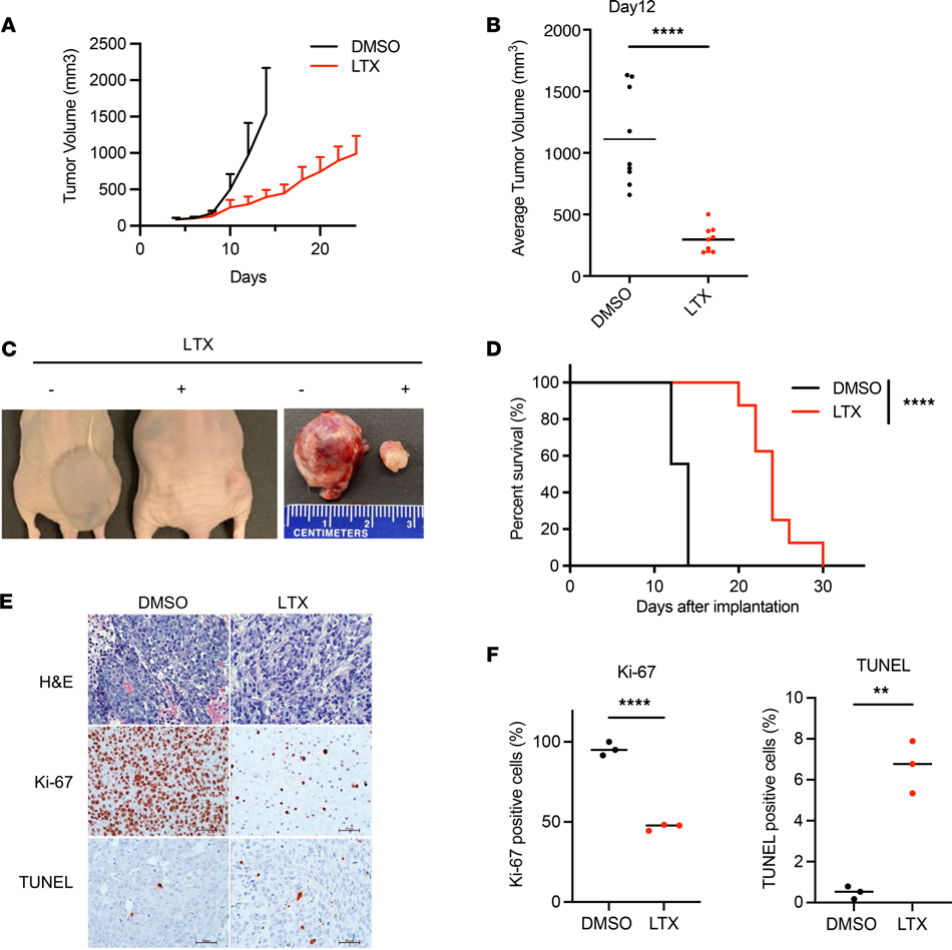
Inhibition of de novo purine synthesis by LTX inhibits MLL4 mutant tumor growth. Tumor development after inoculation of 4 × 10^6^ of HCT116 cells into nude mice. Mice with HCT116 s.c. tumors were treated with either vehicle (DMSO, *n =* 9) or LTX (25 mg/kg, *n =* 9) daily for 7 days. (**A**) Growth plots for s.c. tumors in each treatment group are shown with mean tumor volumes (mm^3^) and upper SD. (**B**) Dot plot representation of s.c. tumor volume in mice at day 12 after tumor cell injection. Unpaired *t* test values for comparisons between DMSO and LTX treatment: ****P < 0.0001 with unpaired *t* test. DMSO, *n =* 9, LTX (25 mg/kg), *n =* 9. (**C**) Photographs of nude mice (left) in which HCT116 cells were inoculated into the right flank and s.c. tumors taken from these mice (right). (**D**) Animal survival at the indicated days after inoculation. Log-rank test was used for comparisons between DMSO and LTX treatment: ****P < 0.0001 with Log-rank (Mantel-Cox) test. (**E**) H&E staining and IHC showing the proliferation and apoptosis in tumors with Ki-67 and TUNEL staining. (**F**) The signals for Ki-67 and TUNEL are quantified and shown in the bar plots. DMSO, *n =* 3, LTX (25 mg/kg), *n =* 3. **P < 0.01, ****P < 0.0001 with unpaired *t* test.

Based on the biological effects of LTX in vitro, we hypothesized that LTX treatment would suppress MLL4 mutant tumor growth in vivo in different xenograft models. To determine the antitumor activity of LTX in MLL4-mutant tumors, we s.c. implanted HCT116 cells into the right flank of mice and treated the mice with LTX (25 mg/kg) or vehicle control (DMSO) i.p. when tumor size reached 100 mm^3^ (day 6 after implantation). Mice were euthanized when the tumor size reached 1,000 mm^3^. LTX treatment significantly inhibited the s.c. tumor growth (*P <* 0.0001, Figure 7, A–C) and extended the survival of HCT116 s.c. xenograft recipient mice compared to the control treatment group (*P <* 0.0001, Figure 7D). Cell proliferation was significantly decreased by LTX treatment, accompanied by enhanced apoptosis (Figure 7, E and F). We next decreased the dosage of LTX to 15 mg/kg and compared the tumor growth of HT55 (MLL4 WT) and HCT116 (MLL4 mutant) colorectal cancer cell xenografts (Supplemental Figure 14). HT55 xenograft tumors were almost completely resistant to LTX (Supplemental Figure 15, A–D), while HCT116 xenograft tumors maintained the sensitivity to LTX even at the lower dose (Supplemental Figure 15, E–H), indicating that MLL4 mutation status may be useful for patient stratification and could guide treatment plan decisions for cancer patients.

## Discussion

**Figure 8:**
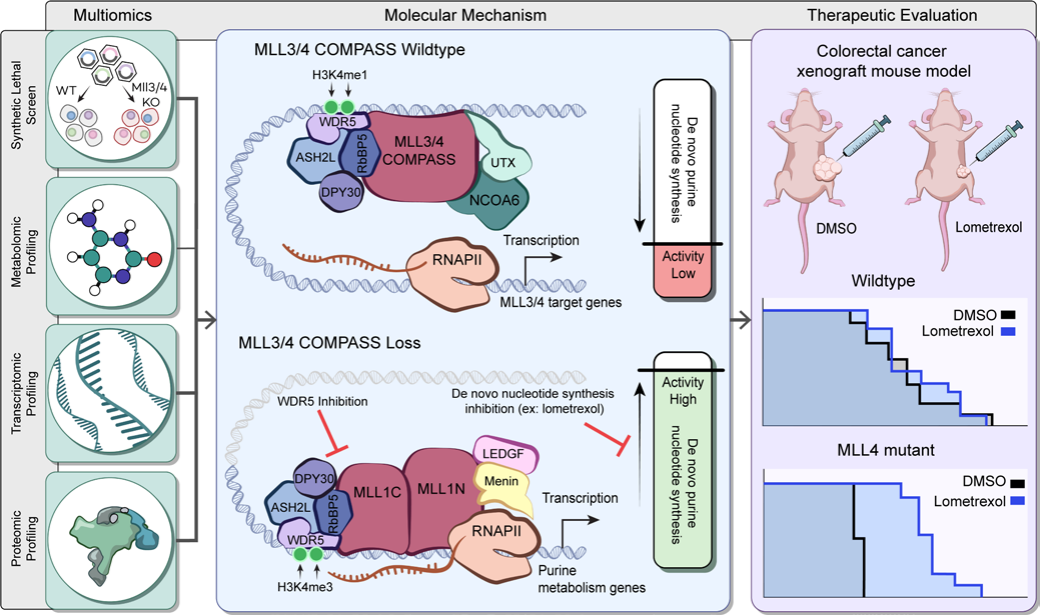
The roadmap of molecular changes and cellular phenotypes when cells lose MLL3/4-COMPASS. To identify targetable cellular vulnerabilities that arise when MLL3/4 are compromised, a variety of multiomics approaches were utilized including a synthetic lethality screen and metabolic, transcriptomic, and proteomic profiling. In addition to the downregulation of MLL3/4 target genes, the loss of MLL3/4 also leads to the activation of MLL1/COMPASS, which drives the upregulated expression of de novo purine metabolism genes. This upregulation was found to be reversible by the specific inhibition of interaction between MLL1 and the cofactor WDR5. These findings led to the therapeutic evaluation of the de novo purine nucleotide synthesis pathway targeting in MLL4 mutant colorectal cancer using lometrexol, a specific inhibitor of the purine synthesis enzyme GART. This promising approach may offer new hope for patients with MLL4 mutant colorectal cancer.

Our study combined a CRISPR dropout screen with metabolomics, transcriptomics, and proteomics approaches, presenting evidence of direct link between epigenetic alteration and metabolic dependency shift (Figure 8). We demonstrated that mESCs depleted of MLL3/4 acquired higher demand for purine nucleotide synthesis and elevated levels of purine metabolism factors (Figure 8). De novo nucleotide synthesis is usually activated in rapidly proliferating cells in response to their enhanced demand for nucleotides to support RNA and DNA synthesis, and cancer cells also tend to increase use of de novo nucleotide synthesis pathways (17). The observation that MLL3/4-KO cells have elevated nucleotide synthesis activity, therefore, suggests a cellular state shift consistent with the impairment of cell fate transition previously observed in the MLL4-deficient condition (10, 11).

Recurrent mutations in the genes encoding MLL3 (*KMT2C*) and MLL4 (*KMT2D*) are frequently found in a broad spectrum of cancers, and some are thought to behave as oncogenic drivers (8, 13, 29, 30). We and other groups have demonstrated that some of these mutations may act as major mediators in the pathogenesis of breast cancer (30), bladder cancer (27, 31, 32), prostate cancer (33), pediatric brain cancer medulloblastoma (34–36), and non-Hodgkin’s lymphoma (30, 37–40). In Kabuki syndrome, a multisystem disorder resulting in developmental abnormalities caused by mutations in *KMT2D* and *KDM6A* genes, approximately 60% of *KMT2D* mutations cause protein truncation resulting in loss of function (14–16). Although the occurrence of recurrent mutations affecting MLL4 has been well documented in solid tumors, hematological malignancies, and Kabuki syndrome, the mechanisms by which MLL4 mutation lesions drive oncogenesis and disease progression remain elusive. Recent exome sequencing and whole-genome sequencing studies revealed that MLL3/4 and UTX mutations are not only found in tumor tissues but are also present in normal tissues such as esophagus (41, 42), endometrial epithelium (43), urothelium (27, 32), skin, and lung (44). This indicates that loss of function due to MLL3/4 COMPASS mutations may lead to macroscopic clonal expansion to further drive tumorigenesis when other key tumor suppressors are comutated. Our study showed an emergent metabolic dependency caused by MLL3/4 loss. This finding provides insight into how clonal expansion may occur and suggests that interventions of metabolic inhibition at early stages might be used to prevent clonal expansion. It is worth investigating whether mutations of MLL3/4 and UTX may elicit broader changes in gene expression that favor cell proliferation or activate proliferative signaling, further inducing metabolic programming. It is also worth determining whether these effects are dosage dependent for MLL3 and MLL4, as most of the loss-of-function mutations in patient tumor samples are heterozygous. We also noticed that the presence of multiple missense mutations may contribute to the abnormal protein functions, such as the mutations in DLD1. MLL4 mutations occur throughout the whole protein, and mutations may elicit different outcomes depending on the different domains they affect. Truncation mutations frequently result in the loss of enzymatic activity because the SET/postSET domains are located at the C-terminus of the protein. Other missense mutations occurring within the critical PHD fingers or other domains may also contribute to abnormal protein functions, due to as-yet-unknown mechanisms. One possibility is the compromised H4K16ac reader function, as a previous study showed that the PHD6 finger of MLL4 (MLL4-PHD6) is a selective reader of the epigenetic modification H4K16ac (45).

A recent study reported that MLL4/UTX/COMPASS is activated when the Menin–MLL1 interaction is disrupted in MLL-rearranged leukemia (46). We observed a similar compensatory effect in which MLL1/COMPASS drives the upregulated expression of genes, including those related to purine metabolism, as MLL1-WDR5 inhibition could partially rescue the altered gene expression in MLL3/4-KO mESCs (Figure 5 and Supplemental Figure 8). The internal equilibrium between COMPASS methyltransferases may dictate the transcriptional output in different settings. Although the effects of Menin-MLL1/2 inhibition did not significantly differ between WT and MLL3/4-KO cells in terms of cell viability, MLL3/4-KO cells exhibited increased sensitivity to MLL1-WDR5 inhibition, suggesting that the catalytic activity of MLL1 is essential to induce the expression of purine metabolism genes. MLL1 inhibition could then be an alternative approach to treatment in MLL4-mutated cancers, a possibility that will be evaluated in our future study. In summary, the effects of MLL1/COMPASS compensation due to MLL3/4 loss represent another layer of gene expression control and contribute to the metabolic phenotype.

MLL4 has also been shown to suppress glycolytic genes in lung tissue, and MLL4 deficiency confers glycolytic vulnerabilities in lung cancer (47) and melanoma (48). However, another study using MEFs and skin fibroblasts showed that MLL4-KO cells display features of reduced mitochondrial oxygen consumption rate and glycolytic flux (49). The disparity between studies may arise from distinct sets of genes regulated by MLL4 in tissues with different origin or enhancer activation. Nevertheless, what can be taken from our study and these previous studies is that MLL4 impacts multiple aspects of metabolism, including nucleotide metabolism, glycolysis, and mitochondrial metabolism, supporting the potential use of targeted metabolic inhibitors such as LTX to suppress MLL4 mutant cancer cell growth. Interestingly, we have noticed a consistent downregulation of mitotic genes such as *PLK1* and *AURKA* after LTX treatment, leading us to define a LTX-responsive mitotic gene signature set. Indeed, our RNA-Seq results also echo those of a previous study that proposed the use of aurora kinase (*AURKA*) inhibitors for MLL4-mutant cancer (50).

The enhanced purine synthesis flux in MLL3/4-KO t cells was consistently accompanied by increased sensitivity to purine nucleotide synthesis inhibition via LTX treatment (Figure 3). We also demonstrated that MLL4-mutant human colorectal cancer cell lines are selectively sensitive to LTX. Metabolic therapies targeting nucleotide and amino acid metabolism have been widely tested for a variety of cancers (51), and multiple nucleotide synthesis inhibitors have been used in clinical trials (52). Our results indicate that further investigation to determine if mutations affecting MLL3/4 COMPASS can predict a higher sensitivity or response to these nucleotide synthesis inhibitors will be critical for personalizing and enhancing patient treatments for cancers and developmental diseases. Overall, we believe that our study provides insight into the identification of novel therapeutic targets and approaches exploiting regulatory reprogramming of metabolic pathways in human cancer that may be extended into future clinical translation.

## Methods

### ESC culture, shRNA knockdown, and CRISPR/Cas9-guided gene editing.

FHC, Caco2, SW1417, HT55, SW480, DLD1, HCT116, and RKO cells were purchased from ATCC. CAL51 cells were purchased from DSMZ. FHC cells were cultured with DMEM/F12 with 25 mM HEPES (Thermo Fisher Scientific), 10 ng/mL choleratoxin (Sigma-Aldrich), 0.005 mg/mL insulin (Lonza), 0.005 mg/mL transferrin (Sigma-Aldrich), 100 ng/mL hydrocortisone (Sigma-Aldrich), 20 ng/mL hEGF (Sigma-Aldrich), and 10% FBS (Sigma-Aldrich). Caco2 and HT55 were grown in DMEM (Thermo Fisher Scientific) with 20% FBS. SW1417, SW480, HCT116 and RKO were grown in DMEM with 10% FBS. DLD1 were grown in RPMI-1640 (Thermo Fisher Scientific) with 10% FBS**.** V6.5 ESCs were grown in N2B27 medium supplemented with 2 inhibitors (2i) and LIF as described previously (3). The lentiviral constructs containing shRNA against all targets were purchased from Millipore Sigma. shRNA sequences are listed in Supplemental Table 1. Lentiviruses were packaged with psPAX2 and CMV-VSVG in 293T cells. At 24 hours and 48 hours after transfection, culture media was collected, passed through 0.45 μm filters, concentrated with the lenti-X concentrator (Takara Bio) and resuspended in mouse ESC media. ESCs were infected with lentiviruses and selected with puromycin (2 μg/ml) for 6 days before collection for RNA extraction.

For CRISPR knockout of MLL3 and MLL4, mESCs were electroporated with plasmids containing sgRNAs in the px459 backbone, selected with puromycin (2 μg/mL; Thermo Fisher Scientific) for 48 hours, and grown in 2i/LIF medium without puromycin until the cell clones were ready to be picked. gRNA sequences used in this study are listed as follows: MLL4 KO, TGGGGATGGACAGCCCGACG (left), GGTATAATCAATCCGTCCTT (right); MLL3 KO, CATATGCTGTAGGAACCGTA (left), TTGGGACAGGTACGAAAATA (right).

### Compound treatment.

LTX (Cayman Chemical, 18049), WDR5-0103 (SelleckChem, S2184), OICR-9429(SelleckChem, S7833), MI-463 (SelleckChem, S7816), MI-503 (SelleckChem, S7817), MTX (MedChemExpress, HY-14519), SHIN1 (MedChemExpress, HY-112066), Piericidin A (Cayman Chemical, 15379), Phenformin (Cayman Chemical, 14997), thymidine (Millipore Sigma, T1895-1G), inosine (Millipore Sigma, I4125-1G), and HTX (Cayman Chemical, 22254) were used with concentrations and durations specified for different experiments.

### Antibodies and Western blot.

The following antibodies are used in this study: anti-H3K4me1 (Cell Signaling Technology [CST], 5326), anti-H3K4me2 (generated in house), anti-H3K4me3 (CST, 9751), anti-H3K27ac (CST, 8173), H3K27me3 (CST, 9733), anti-MLL3 NT (generated in house), anti-MLL3 MT (generated in house), anti-MLL4 NT (generated in house), anti-MLL4 CT (generated in house), anti-MLL2 (generated in house), anti-SET1A (generated in house), anti-Menin (CST, 6891), anti-MLL1C (CST, 14197), anti-RBBP5 (Bethyl Laboratories, A300-109A), anti-NCOA6 (Bethyl Laboratories, A300-410A), anti-UTX (CST, 33510), anti-PTIP (Bethyl Laboratories, A300-370A), ASH2L (CST, 5019), H3 Ser10-p (CST 53348), CDT1 (CST, 8064), Cyclin B1 (CST, 12231), Geminin (CST, 52508), Cyclin E1 (CST, 20808), Cyclin A2 (CST, 91500), p-cdc2 (CST, 4539), PARP (CST, 9542) Caspase3 (CST, 9662), anti-GART (Santa Cruz Biotechnology, sc-166447), anti-PAICS (Proteintech, 12967-1-AP), Hsp90 (Santa Cruz Biotechnology, sc-13119), and anti-β-tubulin (Developmental Studies Hybridoma Bank, E7). Western blot analysis was performed as previously described (53).

### Genome-Scale CRISPR-Cas9 knockout (GeCKO) screening.

The mouse Brie CRISPR knockout pooled library was a gift from David Root and John Doench (Addgene 73633, Broad Institute of MIT and Harvard, Boston) (54). A total of 6.3 × 10^7^ WT or MLL3/4-KO cells were infected with the library at an MOI of 0.5 to ensure that most cells received only 1 genetic perturbation, and that the sgRNA library was well represented in the pool of cells for infection. WT and MLL3/4 knockout mouse ES cells were harvested on day 3 as baseline and selected with puromycin (2 μg/mL) for 15 or 21 days before harvesting. Genomic DNA was extracted, and the library was constructed. After sequencing the library, data analyses were performed with MAGeCK RRA algorithm or MLE module (18). Data visualization was performed with MAGeCKFlute (55).

### NGS data processing.

RNA-Seq and ChIP-Seq samples were sequenced with Illumina NovaSeq technology, and output data were processed with bcl2fastq. Sequence quality was assessed using FastQC v 0.11.2 (56), and quality trimming was done using Trimmomatic (57). RNA-Seq and ChIP-Seq reads were aligned to the mm9 or hg38 genome using TopHat v2.0.9 (58) and Bowtie v0.12.9 (59), and only uniquely mapped reads with a 2-mismatch threshold were considered for downstream analysis. Gene annotations from Ensembl 67 and Ensembl 78 were used. Output bam files were converted into bigwig track files to display coverage throughout the genome (in RPM) using the GenomicRanges package (60) as well as other standard Bioconductor R packages.

### RNA-Seq analysis.

Gene count tables were constructed using HTseq (61) with Ensembl gene annotations and used as input for edgeR 3.0.8 (62). Genes with Benjamini-Hochburg adjusted *P* values less than 0.01 were differentially expressed. Batch effects were removed using ComBat-Seq on the raw read counts (63). Pathway analysis was performed with Metascape (64).

### ChIP-Seq analysis.

5 × 10^7^ cells were used for each ChIP assay and performed as previously described (65). Peaks were called with MACS v1.4.2 using default parameters (66). Peak annotation, pathway analysis, and visualization were performed with ChIPseeker (67). Metaplots were generated using ngsplot (68). Bedtools was used to determine the raw counts at the merged peaks (69). Using in-house perl scripts, raw counts at each peak were converted to RPKM values with total library counts, and log_2_ fold change values between conditions were computed with these normalized values. Nearest genes were identified using in-house perl scripts based on distances between peak centers and TSSs.

### Hi-C and data processing.

Hi-C samples were prepared with the Arima Hi-C kit according to the manufacturer’s instruction. The adapters were trimmed from the Hi-C raw FASTQ files and the trimmed files were mapped against the mm9 mouse reference genome using the runHi-C pipeline. Specifically, the Burrows-Wheeler Aligner was used for the FASTQ file alignment and aligned reads were filtered to remove low quality reads and PCR duplicates. Aligned and filtered reads were then paired on the basis of read pairs and filtered to retain fragments that contain ligations of at least 2 different restriction fragments. These paired reads were then binned at 5-kb resolution.

### Metabolomics study.

The metabolomics study was performed by BIDMC Mass Spectrometry Facility at Beth Israel Deaconess Medical Center. For global steady state metabolomics, cells were grown to approximately 80% confluency and washed with respective medium. Fresh medium was added 2 hours before metabolite collection. For tracing studies, cells were washed with metabolite-free medium and medium containing 4 mM ^15^N-glutamine (Chembridge Isotope Laboratories, NLM-557-1) or 100 μM ^13^C-HTX (Chembridge Isotope Laboratories, CLM-8042-0.01) was added 1 hour before metabolite collection. To collect metabolites, cells were fixed in 80% HPLC-grade methanol in LC-MS water and kept at –80°C for 15 minutes. Next, cells were scraped off the plates on dry ice and transferred to 10 mL conical tubes. Scraped cells were centrifuged, and supernatant (extract) and a second methanol wash were pooled. Extracts were completely dried with Nitrogen gas N-EVAP. Cell pellets were resuspended in 8M urea and a Bradford assay was used to quantify protein concentration for normalization purposes.

### TMT proteomics study.

TMT study was performed by the Thermo Fisher Scientific Center for Multiplexed Proteomics at Harvard Medical School. Samples were prepared in 0.5 mL lysis buffer (8M Urea, 200mM EPPS, pH 8.5, Protease & phosphatase inhibitors). Protein quantification was performed using the micro-BCA assay from Pierce. After protein quantification, lysates were immediately reduced with TCEP and alkylated with iodoacetamide. Approximately 300μg of each sample was precipitated using methanol/chloroform. Digestion was performed using LysC and trypsin. Approximately 100 μg of each sample was labeled with 6 TMT10-plex reagents. A small aliquot of each sample was combined and analyzed by LC-MS2 to evaluate labeling efficiency and mixing ratios. Peptide N terminal ends were labeled >99% by TMT reagents. Samples were combined in full, desalted, and fractionated by HPLC bRP. The sample was fractionated and divided into 2 sets: Set 1 consisted of 12 fractions, each fraction in this set was made up of the orange numbers for an entire column, e.g., 1, 25, 49, 73; Set 2 consisted of 12 fractions from the black numbers for an entire column. One complete set (12 fractions) from HPRP was analyzed on an OrbitrapEclipse mass spectrometer using a real time search method. Peptides were separated using a gradient from 5% to 30% acetonitrile in 0.125% formic acid for over 90 minutes. Peptides were detected (MS1) and quantified (MS3) in the Orbitrap. Peptides were sequenced (MS2) in the ion trap. Peptides were selected for sequencing in MS1 scans. MS2 spectra were used for identifying peptides, and MS3 spectra were used for quantification via TMT reporter ions. mMS2 spectra were searched using the SEQUEST algorithm against a Uniprotcomposite database derived from the mouse proteome, known contaminants, and reverse compliment sequences. Peptide spectral matches were filtered to a 1% FDR using the target-decoy strategy combined with linear discriminant analysis. Proteins were quantified only from peptides with a summed SN threshold of ≥100. Quantified protein and peptide numbers did not include contaminant or reverse sequence identifications.

### Xenograft studies.

Six-week-old female athymic mice (nu/nu genotype, BALB/c background) were purchased from Envigo and housed under aseptic conditions. HT55 or HCT116 cells were implanted into the flank of athymic mice as previously described (70). Briefly, 4 × 10^6^ cells, in 0.4 mL of cell culture media with matrigel (BD Bioscience) at 1:1 ratio, were injected in the right flank of mice under anesthetization by isoflurane. Mice were randomly assigned to vehicle (DMSO, *n =* 9) and LTX treatment (25 mg/kg for 7 days, *n =* 9) groups when the size of tumor reached at 100 mm^3^ (day 6 after implantation). The tumor sizes were measured on alternate days and the mice were euthanized when the tumor size reached 1,000 mm^3^. LTX used in the animal studies was custom synthesized by Enamine Ltd.

### Statistics.

All data were obtained from at least 3 replicates and were expressed as mean ± SD, as indicated in the figure legends. A 2-tailed Student’s *t* test was applied for statistical analysis between 2 groups. The survival information for the mice was summarized by the Kaplan-Meier plots, and the difference was analyzed by the log-rank (Mantel-Cox) test. The statistical analysis was performed using GraphPad Prism 9. *P* values of less than 0.05 were considered significant.

### Study approval.

All animal procedures were approved by the IACUC at Northwestern University and performed in accordance with the IACUC guidelines.

### Data and materials availability.

Next-generation sequencing data sets have been deposited at Gene Expression Omnibus (GEO) with accession number GSE200120. All data are available in the main text or the materials.

## Author contributions

ZZ, KC, and AS conceptualized the project. ZZ, ETB, KC, FY, NSC, RH, IBS, and AS developed the methodology. ZZ, KC, JW, CNP, JMZ, YI, QW, and KJ performed the investigations. ZZ, QW, RH, and IBS were responsible for visualization. AS acquired funding for the project and supervised the project. ZZ and AS wrote the original draft of the manuscript. ZZ, SRG, NSC, RH, IBS, and AS reviewed and edited the manuscript.

## Supplementary Material

Supplemental data

## Version 1 05/30/2023

In-Press Preview

## Version 2 07/03/2023

Electronic publication
