# Supplementary material for: Therapeutic targeting of metabolic vulnerabilities in cancers with MLL3/4-COMPASS epigenetic regulator mutations
Source: J Clin Invest. 2023 Jul 3;133(13):e169993. doi: 10.1172/JCI169993 (PMC10313365; doi:10.1172/JCI169993)
Supplement: Supplemental data [file jci-133-169993-s009.pdf]

Supplementary Materials for

Therapeutic targeting of metabolic vulnerabilities in cancers with MLL3/4-COMPASS epigenetic regulator mutations

Authors:

Zibo Zhao[1,2], Kaixiang Cao[1,2], Jun Watanabe[1,3], Cassandra N. Philips[1,2], Jacob M. Zeidner[1,2], Yukitomo Ishi[1,3], Qixuan Wang[1,2], Sarah R. Gold[1,2], Katherine Junkins[1,2], Elizabeth T. Bartom[1,2], Feng Yue[1,2], Navdeep S. Chandel[1,2,3,4], Rintaro Hashizume[1,3], Issam Ben-Sahra[1,2], and Ali Shilatifard[1,2*]

Affiliations:

[1]Department of Biochemistry and Molecular Genetics, Northwestern University Feinberg School of Medicine, 320 E. Superior St., Chicago, IL 60611, USA

[2]Simpson Querrey Center for Epigenetics, Northwestern University Feinberg School of Medicine, Chicago, IL 60611, USA

[3] Robert H. Lurie NCI Comprehensive Cancer Center, Northwestern University Feinberg School of Medicine, Chicago, IL 60611, USA

[4] Department of Medicine, Northwestern University Feinberg School of Medicine, Chicago, IL 60611, USA

The authors have declared that no conflict of interest exists.

*Correspondence and proofs should be sent to the following address:
Ali Shilatifard
Department of Biochemistry and Molecular Genetics
Northwestern University Feinberg School of Medicine
Simpson Querrey 7th Floor
303 E. Superior St.
Chicago, IL 60611, USA
Email: ASH@northwestern.edu

The PDF file includes:

Supplemental Figure 1 to 15

Supplemental Table 1

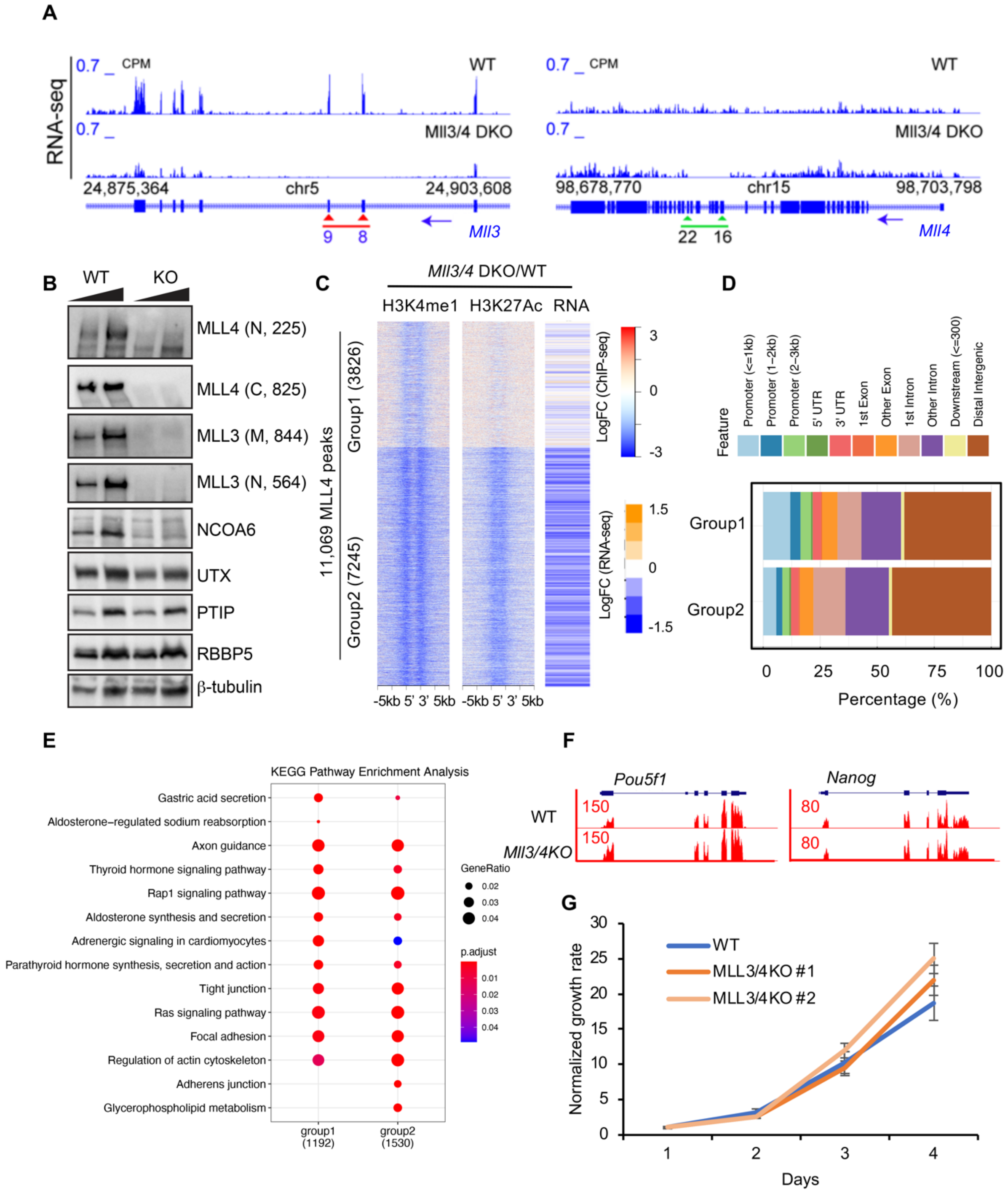
A
RNA-seq
0.7 CPM
WT
Mll3/4 DKO
24,875,364
chr5
24,903,608
9
8
Mll3
98,678,770
chr15
98,703,798
22
16
Mll4
B
WT
KO
MLL4 (N, 225)
MLL4 (C, 825)
MLL3 (M, 844)
MLL3 (N, 564)
NCOA6
UTX
PTIP
RBBP5
β-tubulin
C
Mll3/4 DKO/WT
H3K4me1
H3K27Ac
RNA
11,069 MLL4 peaks
Group1 (3826)
Group2 (7245)
LogFC (ChIP-seq)
LogFC (RNA-seq)
-5kb 5' 3' 5kb
D
Feature
Promoter (<=1kb)
Promoter (1−2kb)
Promoter (2−3kb)
5' UTR
3' UTR
1st Exon
Other Exon
1st Intron
Other Intron
Downstream (<=300)
Distal Intergenic
Group1
Group2
Percentage (%)
E
KEGG Pathway Enrichment Analysis
Gastric acid secretion
Aldosterone−regulated sodium reabsorption
Axon guidance
Thyroid hormone signaling pathway
Rap1 signaling pathway
Aldosterone synthesis and secretion
Adrenergic signaling in cardiomyocytes
Parathyroid hormone synthesis, secretion and action
Tight junction
Ras signaling pathway
Focal adhesion
Regulation of actin cytoskeleton
Adherens junction
Glycerophospholipid metabolism
group1 (1192)
group2 (1530)
GeneRatio
p.adjust
F
Pou5f1
Nanog
WT
Mll3/4KO
G
Normalized growth rate
WT
MLL3/4KO #1
MLL3/4KO #2
Days

Supplemental Figure 1. Characterization of MLL3 and MLL4 double knockout mESCs. (**A**) RNA-seq track showing the CRISPR strategy to knock out MLL3 and MLL4 by targeting exons 8-9 and exons 16-22, respectively. (**B**) Western blot showing the successful knockout of MLL3 and MLL4 with β-tubulin and the pan-COMPASS subunit RBBP5 as internal controls. Other subunits specific to MLL3/4 COMPASS including NCOA6, UTX, and PTIP are also shown with decreased signals in MLL3/4 KO cells. N, N-terminus; M, middle region; C, C-terminus. (**C**) H3K4me1 and H3K27Ac ChIP-seq was performed in WT and MLL3/4 KO cells. K-means clustering (k=2) separated the previously defined MLL4 peaks (*1*) into two groups based on the log2 fold change in H3K4me1 and H3K27Ac ChIP-seq signals. In the meta-analysis shown, H3K4me1 and H3K27Ac reduction coincides with strongly reduced expression of genes at Group2 MLL4 peaks in MLL3/4 KO cells. (**D**-**E**) Feature distribution of Group1 and Group2 MLL4 peaks (**D**) and the KEGG pathway enrichment analysis of Group1 and Group2 genes (**E**) using the ChIPseeker package(*2*). (**F**) RNA-seq track showing the *Pou5f1* and *Nanog* gene expression in WT and MLL3/4 KO cells. (**G**) Cell growth rates of WT and MLL3/4 KO cells after sustained passaging. Cells were seeded in 6-well plates at 1x105 cells/mL. Cell number was counted with Beckman Vi-Cell XR Cell Viability Analyzer. Cell numbers were normalized to day 1. n=2.

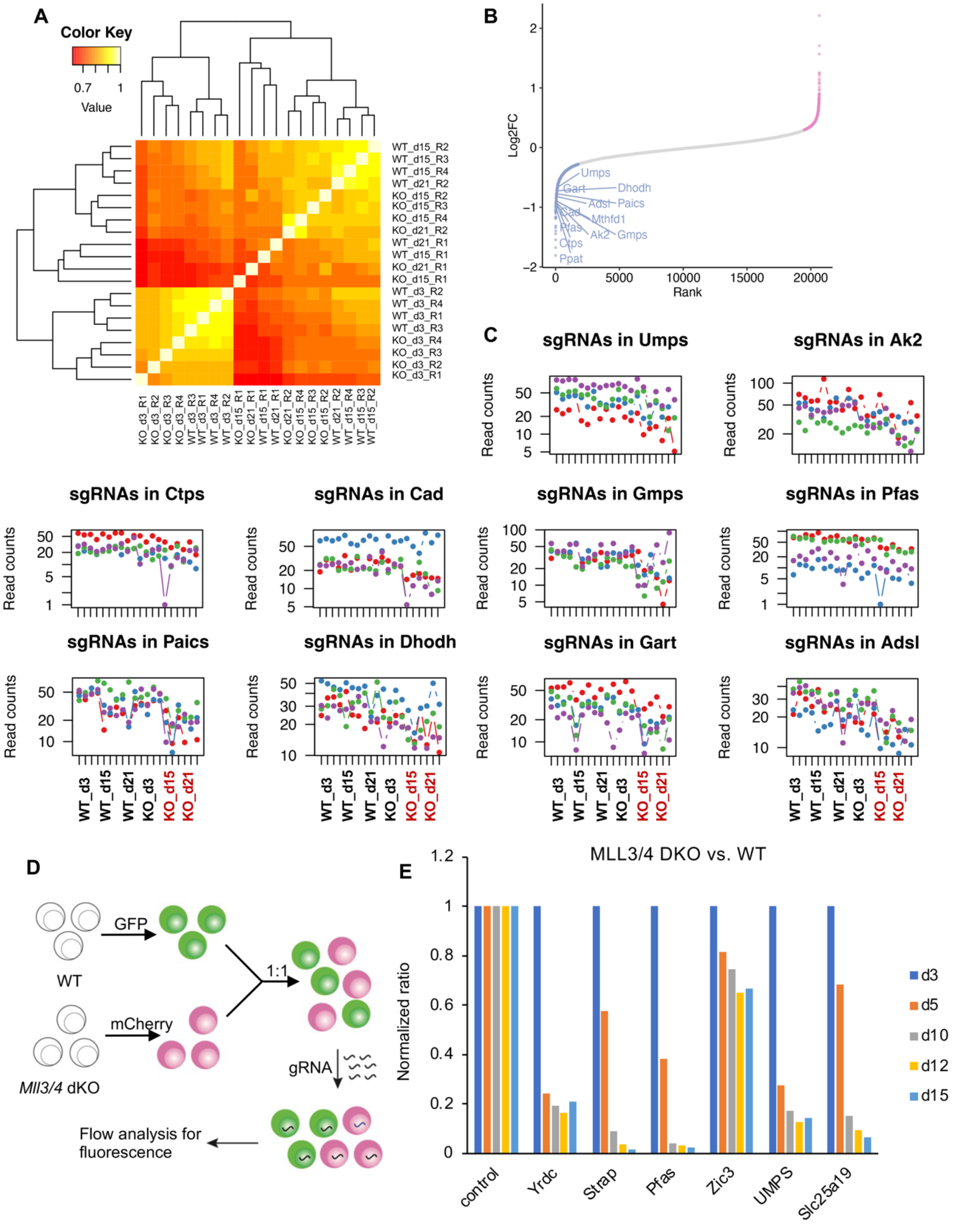
A
Color Key
0.7
1
Value
WT_d15_R2
WT_d15_R3
WT_d15_R4
WT_d21_R2
KO_d15_R2
KO_d15_R3
KO_d15_R4
KO_d21_R2
WT_d21_R1
WT_d15_R1
KO_d21_R1
KO_d15_R1
WT_d3_R2
WT_d3_R4
WT_d3_R1
WT_d3_R3
KO_d3_R4
KO_d3_R3
KO_d3_R2
KO_d3_R1
B
Log2FC
Rank
Umps
Gart
Dhodh
Adsl
Paics
Cad
Mthfd1
Pfas
Ak2
Gmps
Ctps
Ppat
C
sgRNAs in Umps
sgRNAs in Ak2
sgRNAs in Ctps
sgRNAs in Cad
sgRNAs in Gmps
sgRNAs in Pfas
sgRNAs in Paics
sgRNAs in Dhodh
sgRNAs in Gart
sgRNAs in Adsl
Read counts
WT_d3
WT_d15
WT_d21
KO_d3
KO_d15
KO_d21
D
GFP
WT
mCherry
Mll3/4 dKO
1:1
gRNA
Flow analysis for
fluorescence
E
MLL3/4 DKO vs. WT
Normalized ratio
d3
d5
d10
d12
d15
control
Yrdc
Strap
Pfas
Zic3
UMPS
Slc25a19

Supplemental Figure 2. Genome-wide Screen Identifies Purine and Pyrimidine Synthesis Pathways as Essential in MLL3/4 KO mESCs. (**A**) Sample clustering of replicates for different conditions and time points. (**B**) Overall log2FC rank plot with purine/pyrimidine genes highlighted. (**C**) Distribution of sgRNA read counts (normalized) of purine/pyrimidine synthesis genes in WT and KO cells at different time points. (**D**) WT and KO cells were labeled with GFP and mCherry and were mixed at a 1:1 ratio. Mixed cells were infected with sgRNAs, and fluorescence was analyzed with flow cytometry. (**E**) The validation of several negatively selected genes in MLL3/4 KO. Normalized ratio of mCherry/GFP indicating selective sgRNA impact on KO cells.

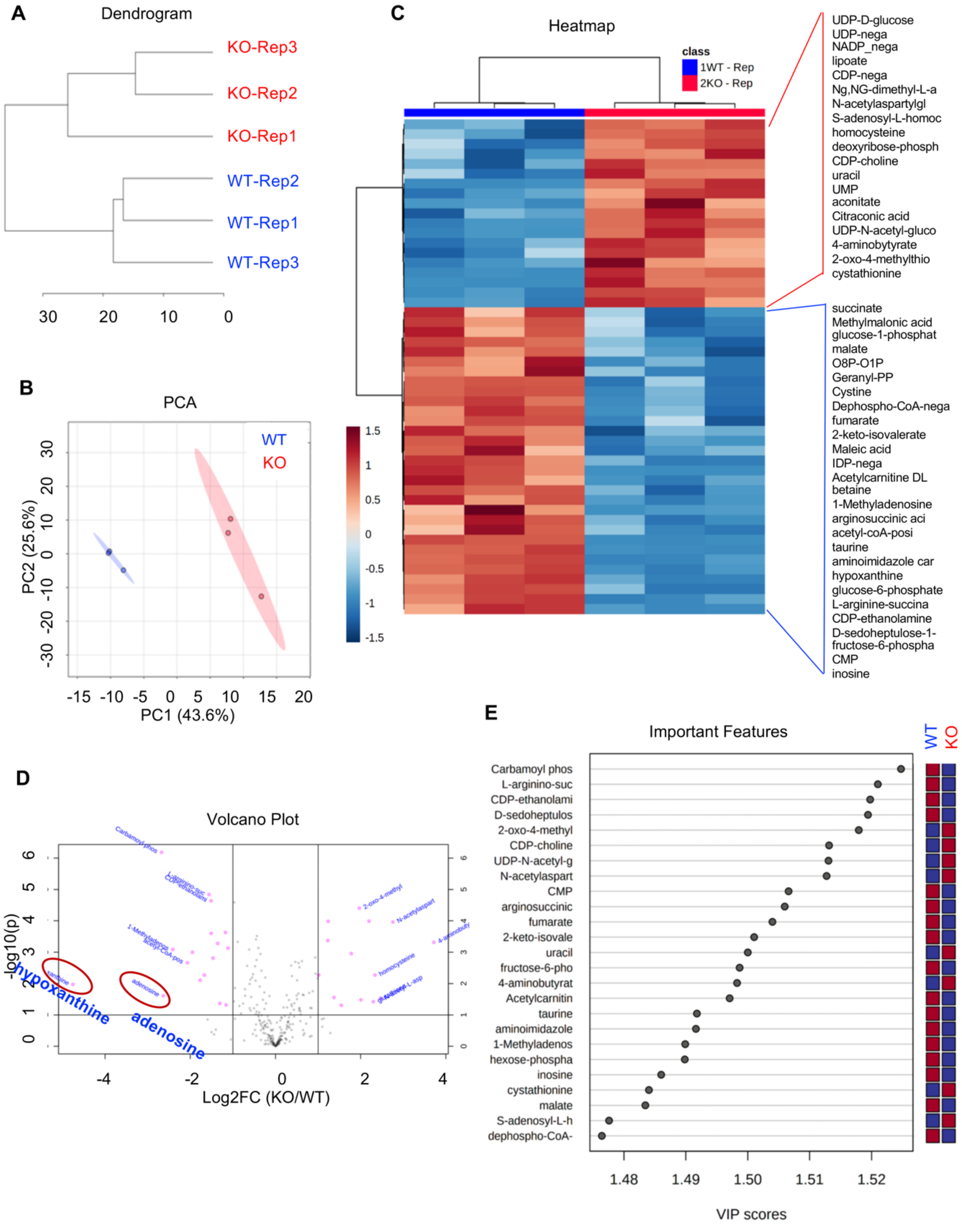
A
Dendrogram
KO-Rep3
KO-Rep2
KO-Rep1
WT-Rep2
WT-Rep1
WT-Rep3
30 20 10 0
B
PCA
WT
KO
PC2 (25.6%)
PC1 (43.6%)
C
Heatmap
class
1WT - Rep
2KO - Rep
UDP-D-glucose
UDP-nega
NADP_nega
lipoate
CDP-nega
Ng,NG-dimethyl-L-a
N-acetylaspartylgl
S-adenosyl-L-homoc
homocysteine
deoxyribose-phosph
CDP-choline
uracil
UMP
aconitate
Citraconic acid
UDP-N-acetyl-gluco
4-aminobytyrate
2-oxo-4-methylthio
cystathionine
succinate
Methylmalonic acid
glucose-1-phosphat
malate
O8P-O1P
Geranyl-PP
Cystine
Dephospho-CoA-nega
fumarate
2-keto-isovalerate
Maleic acid
IDP-nega
Acetylcarnitine DL
betaine
1-Methyladenosine
arginosuccinic aci
acetyl-coA-posi
taurine
aminoimidazole car
hypoxanthine
glucose-6-phosphate
L-arginine-succina
CDP-ethanolamine
D-sedoheptulose-1-
fructose-6-phospha
CMP
inosine
D
Volcano Plot
-log10(p)
hypoxanthine
adenosine
Log2FC (KO/WT)
E
Important Features
WT
KO
Carbamoyl phos
L-arginino-suc
CDP-ethanolami
D-sedoheptulos
2-oxo-4-methyl
CDP-choline
UDP-N-acetyl-g
N-acetylaspart
CMP
arginosuccinic
fumarate
2-keto-isovale
uracil
fructose-6-pho
4-aminobutyrat
Acetylcarnitin
taurine
aminoimidazole
1-Methyladenos
hexose-phospha
inosine
cystathionine
malate
S-adenosyl-L-h
dephospho-CoA-
VIP scores

Supplemental Figure 3. Global Steady State Metabolomics of WT and MLL3/4 KO mESCs. (**A**) Dendrogram of steady state metabolomics for WT and KO cells. (**B**) PC analysis of WT and KO cells steady state metabolomics. (**C**) Hierarchical clustering heatmap showing the differentially abundant metabolites in WT and KO cells. The scale bar represents the Z-score. (**D**) Volcano plot showing the significantly altered metabolites by log10 p value and log2 fold change. (**E**) Important metabolic features in WT and KO cells ranked by VIP scores.

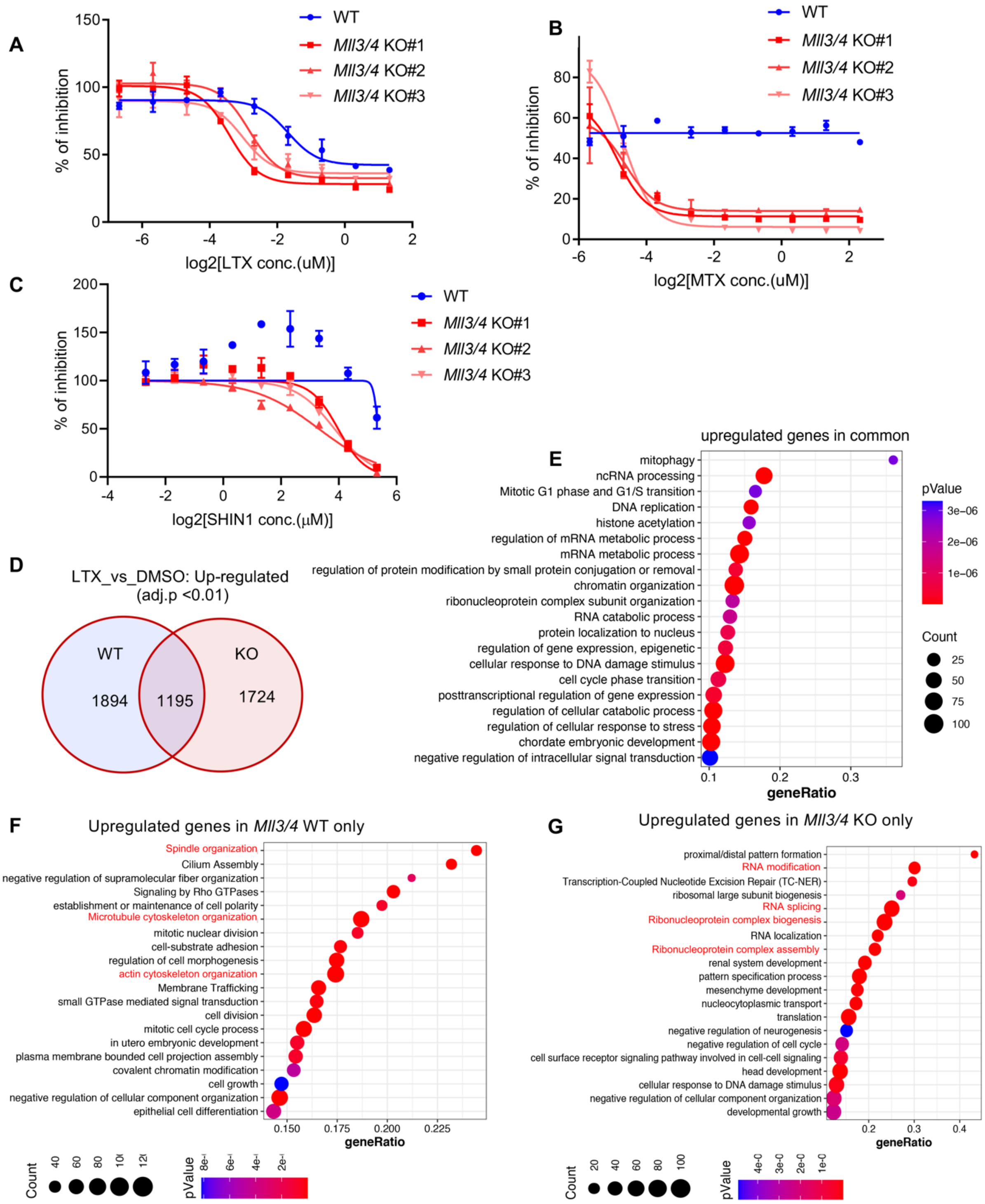
A
150
100
50
0
% of inhibition
-6
-4
-2
0
2
log2[LTX conc.(uM)]
WT
Mll3/4 KO#1
Mll3/4 KO#2
Mll3/4 KO#3
B
80
60
40
20
0
% of inhibition
-6
-4
-2
0
2
log2[MTX conc.(uM)]
WT
Mll3/4 KO#1
Mll3/4 KO#2
Mll3/4 KO#3
C
200
150
100
50
0
% of inhibition
-4
-2
0
2
4
6
log2[SHIN1 conc.(μM)]
WT
Mll3/4 KO#1
Mll3/4 KO#2
Mll3/4 KO#3
D
LTX_vs_DMSO: Up-regulated
(adj.p <0.01)
WT
KO
1894
1195
1724
E
upregulated genes in common
mitophagy
ncRNA processing
Mitotic G1 phase and G1/S transition
DNA replication
histone acetylation
regulation of mRNA metabolic process
mRNA metabolic process
regulation of protein modification by small protein conjugation or removal
chromatin organization
ribonucleoprotein complex subunit organization
RNA catabolic process
protein localization to nucleus
regulation of gene expression, epigenetic
cellular response to DNA damage stimulus
cell cycle phase transition
posttranscriptional regulation of gene expression
regulation of cellular catabolic process
regulation of cellular response to stress
chordate embryonic development
negative regulation of intracellular signal transduction
0.1
0.2
0.3
geneRatio
pValue
3e−06
2e−06
1e−06
Count
25
50
75
100
F
Upregulated genes in Mll3/4 WT only
Spindle organization
Cilium Assembly
negative regulation of supramolecular fiber organization
Signaling by Rho GTPases
establishment or maintenance of cell polarity
Microtubule cytoskeleton organization
mitotic nuclear division
cell-substrate adhesion
regulation of cell morphogenesis
actin cytoskeleton organization
Membrane Trafficking
small GTPase mediated signal transduction
cell division
mitotic cell cycle process
in utero embryonic development
plasma membrane bounded cell projection assembly
covalent chromatin modification
cell growth
negative regulation of cellular component organization
epithelial cell differentiation
0.150
0.175
0.200
0.225
geneRatio
Count
40
60
80
pValue
G
Upregulated genes in Mll3/4 KO only
proximal/distal pattern formation
RNA modification
Transcription-Coupled Nucleotide Excision Repair (TC-NER)
ribosomal large subunit biogenesis
RNA splicing
Ribonucleoprotein complex biogenesis
RNA localization
Ribonucleoprotein complex assembly
renal system development
pattern specification process
mesenchyme development
nucleocytoplasmic transport
translation
negative regulation of neurogenesis
negative regulation of cell cycle
cell surface receptor signaling pathway involved in cell-cell signaling
head development
cellular response to DNA damage stimulus
negative regulation of cellular component organization
developmental growth
0.2
0.3
0.4
geneRatio
Count
20
40
60
80
100
pValue

Supplemental Figure 4. MLL3/4 KO mESCs are more sensitive to purine synthesis inhibition. (**A-B**) WT and MLL3/4 KO cells were treated with 0 – 2.5 μM lometrexol (LTX) (**A**) or 0 – 5 μM methotrexate (MTX) (**B**) for 48 hours. A CellTiter-Glo® luminescent cell viability assay was performed to determine the percentage of cell growth inhibition under these conditions with three different MLL3/4 KO clones included. (**C**) WT and MLL3/4 KO cells were treated with 0 – 40 μM SHIIN1 for 48 hours. An MTT cell viability assay was performed to determine the percentage of cell growth inhibition under these conditions with three different MLL3/4 KO clones included. n=3. (**D**) Venn diagram showing the overlap of genes upregulated upon LTX treatment in WT or KO cells. (**E**) Pathway enrichment analysis of the genes upregulated upon LTX treatment in both WT and KO cells. (**F**) Pathway enrichment analysis of the genes uniquely upregulated upon LTX treatment in WT cells. (**G**) Pathway enrichment analysis of the genes uniquely upregulated upon LTX treatment in MLL3/4 KO cells.

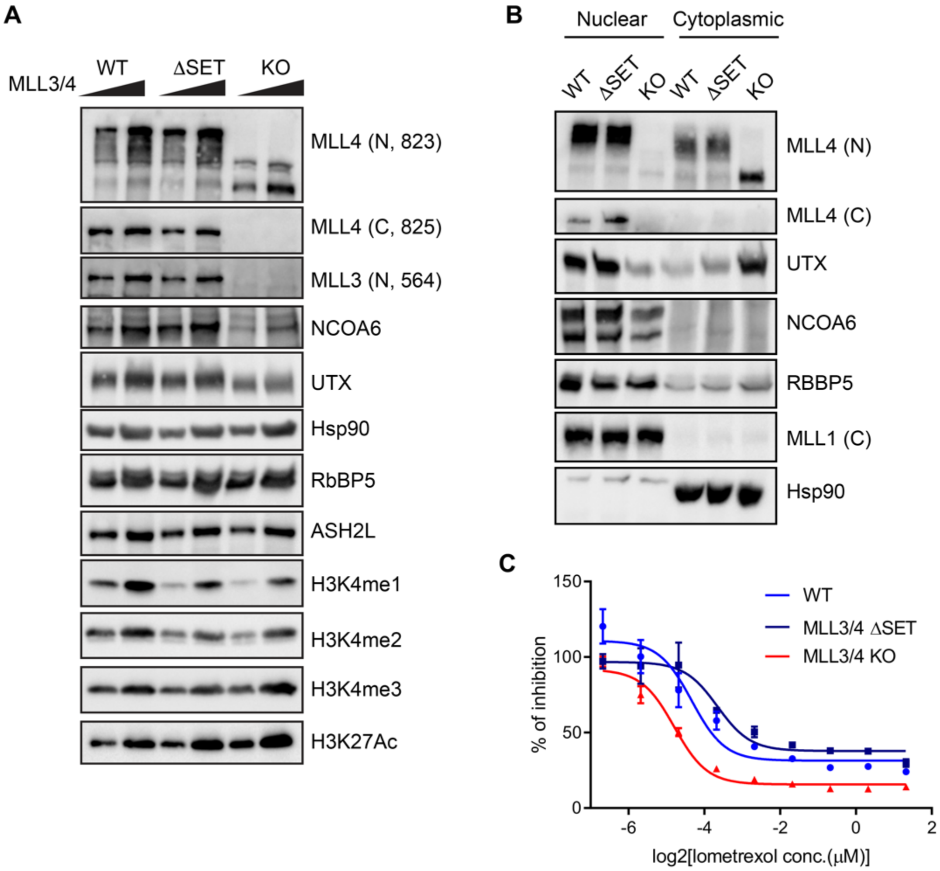
A
MLL3/4
WT
ΔSET
KO
MLL4 (N, 823)
MLL4 (C, 825)
MLL3 (N, 564)
NCOA6
UTX
Hsp90
RbBP5
ASH2L
H3K4me1
H3K4me2
H3K4me3
H3K27Ac
B
Nuclear
Cytoplasmic
WT
ΔSET
KO
WT
ΔSET
KO
MLL4 (N)
MLL4 (C)
UTX
NCOA6
RBBP5
MLL1 (C)
Hsp90
C
WT
MLL3/4 ΔSET
MLL3/4 KO
% of inhibition
150
100
50
0
-6
-4
-2
0
2
log2[lometrexol conc.(μM)]

Supplemental Figure 5. Loss of the MLL3/4 SET domain does not confer increased sensitivity to purine synthesis inhibition. (**A**) Western blot showing MLL4, MLL3, NCOA6, UTX, RbBP5 and ASH2L protein levels in WT, MLL3/4 ΔSET and MLL3/4 KO cells with Hsp90 as the loading control. Bulk levels of histone H3K4me1, H3K4me2, H3K4me3 and H3K27Ac are also shown. N, N-terminus; C, C-terminus. mESCs harboring deletion of the SET domains of both MLL3 and MLL4 (referred to as MLL3/4 ΔSET) were generated in our previous studies (*3*). (**B**) Western blot showing nuclear and cytoplasmic MLL4, UTX, NCOA6 and RBBP5 protein levels after cell fractionation, with MLL1 and Hsp90 serving as nuclear and cytoplasmic loading controls, respectively. (**C**) WT, MLL3/4 ΔSET and MLL3/4 KO cells were treated with 0 – 2.5 μM lometrexol (LTX) for 72 hours. A CellTiter-Glo® luminescent cell viability assay was performed to determine the percentage of inhibition. n=2.

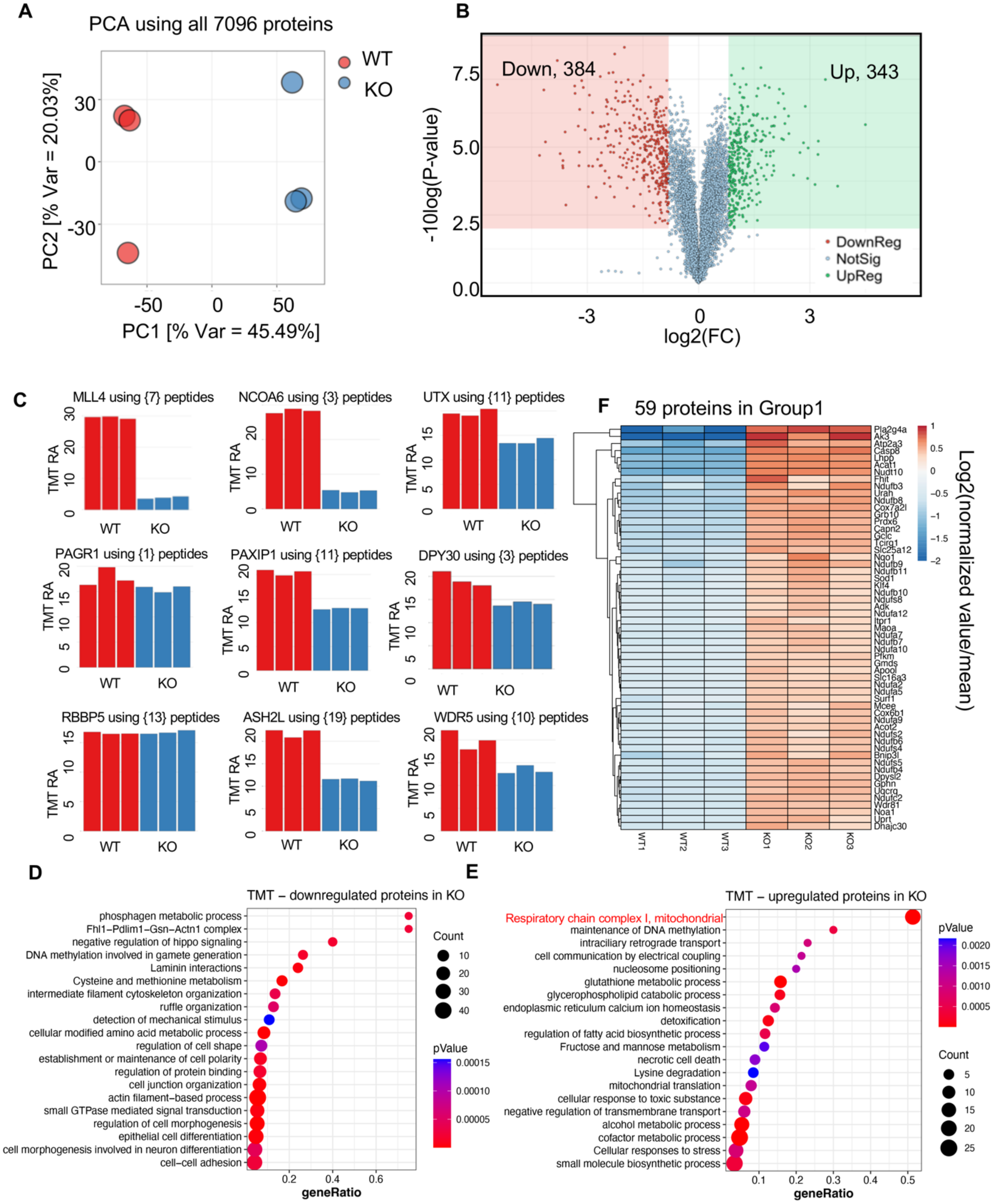
A
PCA using all 7096 proteins
WT
KO
PC2 [% Var = 20.03%]
PC1 [% Var = 45.49%]
30
0
-30
-50
0
50
B
Down, 384
Up, 343
-10log(P-value)
log2(FC)
7.5
5.0
2.5
0.0
-3
0
3
DownReg
NotSig
UpReg
C
MLL4 using {7} peptides
NCOA6 using {3} peptides
UTX using {11} peptides
PAGR1 using {1} peptides
PAXIP1 using {11} peptides
DPY30 using {3} peptides
RBBP5 using {13} peptides
ASH2L using {19} peptides
WDR5 using {10} peptides
TMT RA
WT
KO
F
59 proteins in Group1
Pla2g4a
Ak3
Atp2a3
Casp8
Lhpp
Acat1
Nudt10
Fhit
Ndufb3
Urah
Ndufb8
Cox7a2l
Grb10
Prdx6
Capn2
Gclc
Tcirg1
Slc25a12
Nqo1
Ndufb9
Ndufb11
Sod1
Klf4
Ndufb10
Ndufs8
Adk
Ndufa12
Itpr1
Maoa
Ndufa7
Ndufb7
Ndufa10
Pfkm
Gmds
Apool
Slc16a3
Ndufa2
Ndufa5
Surf1
Mcee
Cox6b1
Ndufa9
Acot2
Ndufs2
Ndufb6
Ndufs4
Bnip3l
Ndufs5
Ndufb4
Dpysl2
Gphn
Uqcrq
Ndufc2
Wdr81
Noa1
Uprt
Dnajc30
WT1
WT2
WT3
KO1
KO2
KO3
1
0.5
0
−0.5
−1
−1.5
−2
Log2(normalized value/mean)
D
TMT − downregulated proteins in KO
phosphagen metabolic process
Fhl1−Pdlim1−Gsn−Actn1 complex
negative regulation of hippo signaling
DNA methylation involved in gamete generation
Laminin interactions
Cysteine and methionine metabolism
intermediate filament cytoskeleton organization
ruffle organization
detection of mechanical stimulus
cellular modified amino acid metabolic process
regulation of cell shape
establishment or maintenance of cell polarity
regulation of protein binding
cell junction organization
actin filament−based process
small GTPase mediated signal transduction
regulation of cell morphogenesis
epithelial cell differentiation
cell morphogenesis involved in neuron differentiation
cell−cell adhesion
Count
10
20
30
40
pValue
0.00015
0.00010
0.00005
0.2
0.4
0.6
geneRatio
E
TMT − upregulated proteins in KO
Respiratory chain complex I, mitochondrial
maintenance of DNA methylation
intraciliary retrograde transport
cell communication by electrical coupling
nucleosome positioning
glutathione metabolic process
glycerophospholipid catabolic process
endoplasmic reticulum calcium ion homeostasis
detoxification
regulation of fatty acid biosynthetic process
Fructose and mannose metabolism
necrotic cell death
Lysine degradation
mitochondrial translation
cellular response to toxic substance
negative regulation of transmembrane transport
alcohol metabolic process
cofactor metabolic process
Cellular responses to stress
small molecule biosynthetic process
pValue
0.0020
0.0015
0.0010
0.0005
Count
5
10
15
20
25
0.1
0.2
0.3
0.4
0.5
geneRatio

Supplemental Figure 6. TMT proteomics profiling identified purine metabolism upregulation in MLL3/4 KO cells. (**A**) TMT proteomics profiling was conducted in WT and MLL3/4 KO cells. PCA plot showing the separation of genotypes by PC1 using all 7096 proteins identified. (**B**) Volcano plot showing all the differentially abundant proteins in WT and KO cells with the cutoff $|FC| > 1.75$, $p.val < 0.01$. Up, 343 proteins; Down, 384 proteins. (**C**) Protein levels of MLL3/4 COMPASS subunits identified and quantified by the TMT approach. (**D**) Pathway enrichment analysis of the downregulated proteins in KO cells in comparison to WT cells. (**E**) Pathway enrichment analysis of the upregulated proteins in KO cells in comparison to WT cells. The mitochondrial respiratory chain complex I group (referred to as Group1 here) was significantly enriched. (**F**) Heatmap showing the 59 proteins in Group1 change in WT and KO cells related to nucleotide metabolic processes.

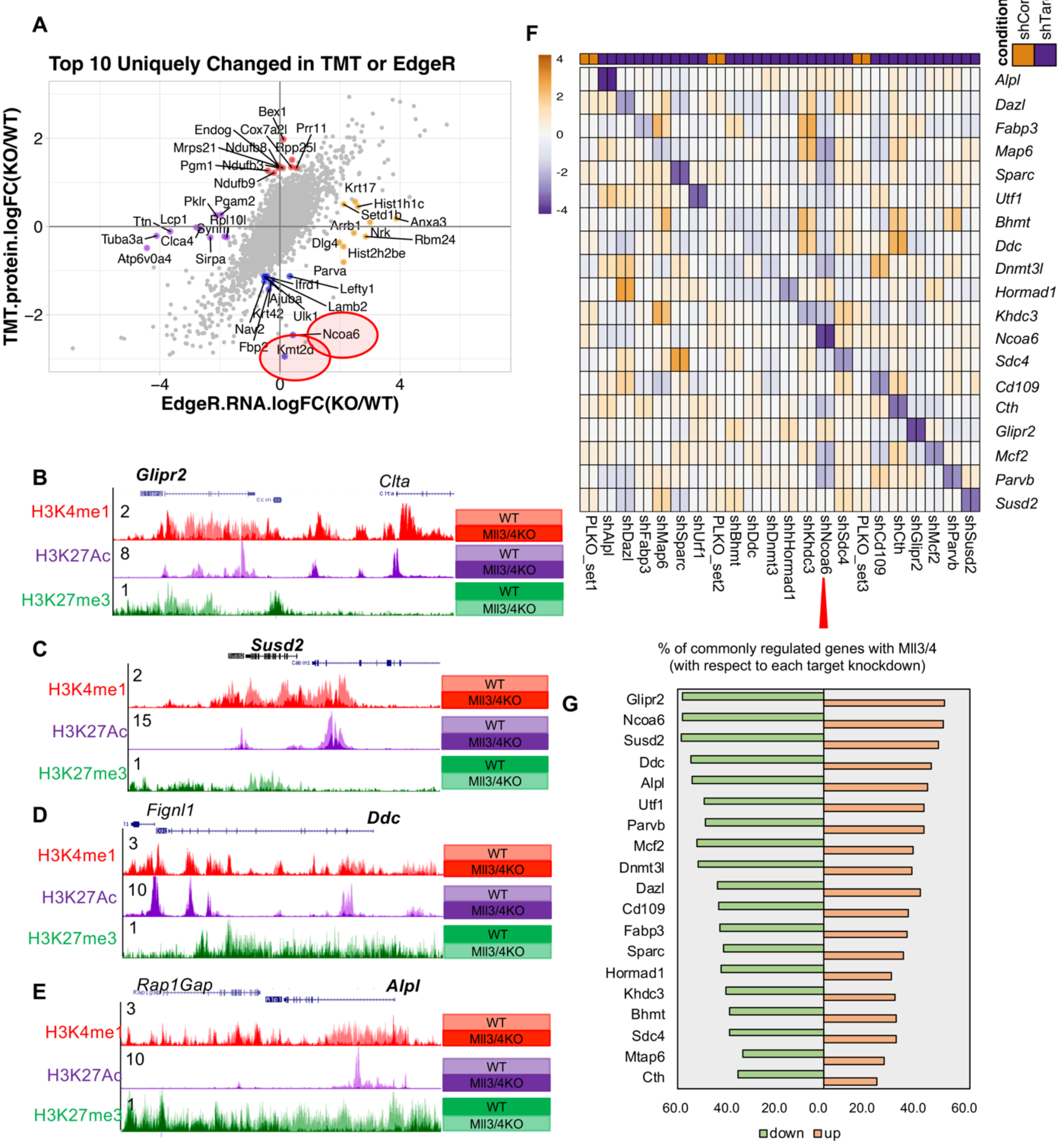
A
Top 10 Uniquely Changed in TMT or EdgeR
TMT.protein.logFC(KO/WT)
EdgeR.RNA.logFC(KO/WT)
Bex1
Endog
Cox7a2l
Prr11
Mrps21
Ndufb8
Rpp25l
Pgm1
Ndufb3
Ndufb9
Krt17
Pklr
Pgam2
Hist1h1c
Setd1b
Ttn
Lcp1
Rpl10l
Arrb1
Anxa3
Syrn
Nrk
Tuba3a
Clca4
Dlg4
Rbm24
Atp6v0a4
Sirpa
Hist2h2be
Parva
Ifrd1
Lefty1
Ajuba
Lamb2
Krt42
Ulk1
Nav2
Ncoa6
Fbp2
Kmt2d
B
Glipr2
Clta
H3K4me1
H3K27Ac
H3K27me3
WT
Mll3/4KO
C
Susd2
D
Fignl1
Ddc
E
Rap1Gap
Alpl
F
condition
shControl
shTarget
Alpl
Dazl
Fabp3
Map6
Sparc
Utf1
Bhmt
Ddc
Dnmt3l
Hormad1
Khdc3
Ncoa6
Sdc4
Cd109
Cth
Glipr2
Mcf2
Parvb
Susd2
PLKO_set1
shAlpl
shDazl
shFabp3
shMap6
shSparc
shUrf1
PLKO_set2
shBhmt
shDdc
shDnmt3
shHormad1
shKhdc3
shNcoa6
shSdc4
PLKO_set3
shCd109
shCth
shGlipr2
shMcf2
shParvb
shSusd2
% of commonly regulated genes with Mll3/4
(with respect to each target knockdown)
G
Glipr2
Ncoa6
Susd2
Ddc
Alpl
Utf1
Parvb
Mcf2
Dnmt3l
Dazl
Cd109
Fabp3
Sparc
Hormad1
Khdc3
Bhmt
Sdc4
Mtap6
Cth
down
up

Supplemental Figure 7. RNA-seq and TMT proteomics profiling integration identified top MLL3/4 target genes. (**A**) The correlation analysis of TMT and EdgeR data identified factors including NCOA6 that were altered at the protein but not RNA levels. (**B-E**) Browser tracks with overlaid view of H3K4me1, H3K27Ac and H3K27me3 ChIP-seq for selected downregulated target genes including *Glipr2* (**B**), *Susd2* (**C**), *Ddc* (**D**), and *Alpl* (**E**). (**F**) Knockdown efficiency of each specific target shown by the z-score heatmap. (**G**) Percentage of genes regulated in common with MLL3/4 for each target knockdown.

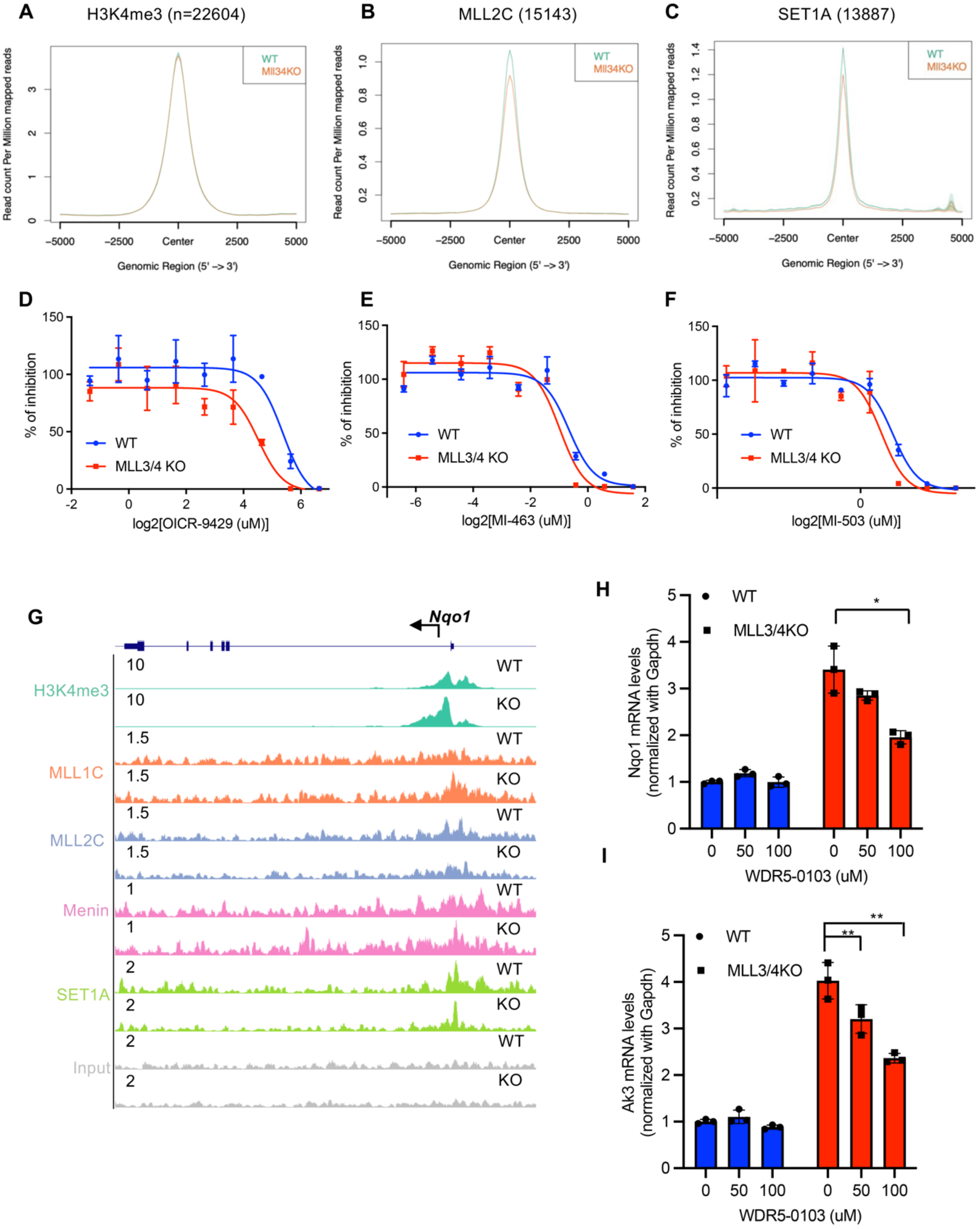
A
H3K4me3 (n=22604)
WT
Mll34KO
Read count Per Million mapped reads
Genomic Region (5' -> 3')
B
MLL2C (15143)
C
SET1A (13887)
D
% of inhibition
WT
MLL3/4 KO
log2[OICR-9429 (uM)]
E
log2[MI-463 (uM)]
F
log2[MI-503 (uM)]
G
Nqo1
H3K4me3
MLL1C
MLL2C
Menin
SET1A
Input
WT
KO
H
Nqo1 mRNA levels (normalized with Gapdh)
WT
MLL3/4KO
WDR5-0103 (uM)
I
Ak3 mRNA levels (normalized with Gapdh)

Supplemental Figure 8. MLL1 compensates the loss of MLL3/4 to activate gene expression. (**A**) H3K4me3 ChIP-seq peaks in both WT and MLL3/4 KO cells were merged and sorted. Average plot showing the H3K4me3 signal in WT and MLL3/4 KO cells centered on all H3K4me3 peaks. n = 22604. (**B**) MLL2C (MLL2 Carboxyl-terminal antibody) ChIP-seq peaks in both WT and MLL3/4 KO cells were merged and sorted. Average plot showing the MLL2C signal in WT and MLL3/4 KO cells centered on all MLL2 peaks. n = 15143. (**C**) SET1A ChIP-seq peaks in both WT and MLL3/4 KO cells were merged and sorted. Average plot showing the SET1A signal in WT and MLL3/4 KO cells centered on all SET1A peaks. n = 13887. (**D-F**) Cell viability of WT and MLL3/4 KO cells in response to OIR-9429 (**D**), MI-463 (**E**) or MI-503 (**F**) treatment. N=3 for each specific concentration in WT and MLL3/4 KO. (**G**) ChIP-seq track showing the chromatin occupancy of H3K4me3, MLL1, MLL2, Menin and SET1A at the *Nqo1* gene locus in WT and MLL3/4 KO cells. (**H**) qRT-PCR showing *Nqo1* (**H**) and *Ak3* (**I**) gene expression in WT and MLL3/4 KO cells treated with 0, 50, or 100 μM WDR5-0103. mRNA levels were normalized to the *Gapdh* internal control. Data are presented as mean ± standard deviation (SD) with n=3. *P < 0.05, **P < 0.01 with unpaired t-test.

A

Sample	WT	MLL3/4 KO
Loop number (10 kb)	10,118	13,695
TAD number (40 kb)	2,514	2,447
A/B Compartment number (100 kb)	10,884/14,178	12,141/12,874

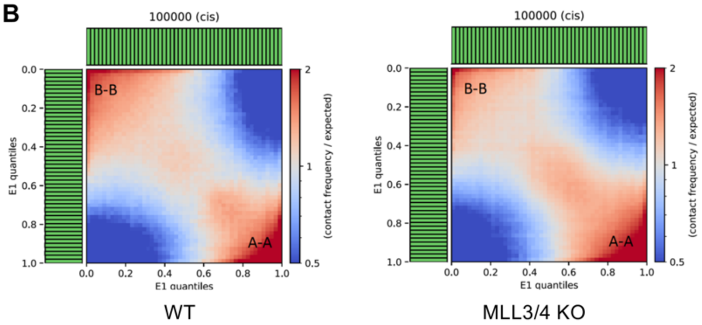

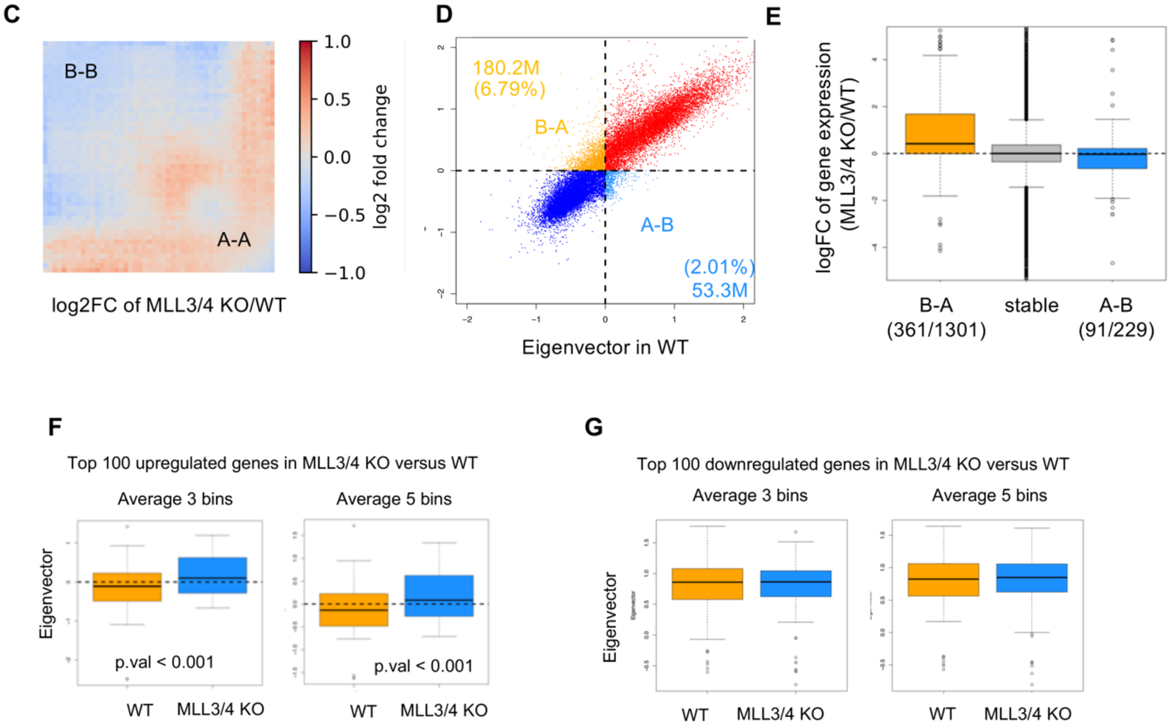

Supplementary Figure 9. Hi-C analysis in WT and MLL3/4 KO mESCs. (**A**) Loop number, TAD (Topologically Associated Domains) number, and A/B compartment occupancy numbers in WT and MLL3/4 KO mESCs. (**B**) Hi-C was performed in WT and MLL3/4 KO mESCs. Compartmentalization saddle plots of Hi-C data for WT and MLL3/4 KO ES cells, created using cooltools (4). Average intra-chromosomal interaction frequencies were normalized by expected interaction frequency and distance. Bins were sorted by eigenvector PC1 values. B-B interactions are located in the upper-left corner of saddle plots and A-A interactions are located in the lower-right corner. (**C**) Saddle plot of Log2FC of MLL3/4 KO versus WT. **(D)** A C-score was calculated for each 100-kb genomic bin to determine its A or B compartmentalization. Compartment switching, decompaction and compaction upon MLL3/4 loss are represented in the scatter plot. Lengths of genomic regions (no. of bins x 100 kb) and percentage of compartment shifts are shown. **(E)** Genes located in B-to-A shifted (B-A), stable and A-to-B shifted (A-B) bins were selected (>= ½ gene length located within the bins). 1301 and 229 genes were found in B-A and A-B group, respectively. Genes with detectable expression levels were further selected (361 for B-A and 91 for A-B) for analysis of the logFC gene expression in KO versus WT, shown in the box plot. (**F-G**) Eigenvector values for the top 100 up (**F**) or down (**G**) regulated genes. "3 bins average" is the average of the 100 kb bin containing the gene's TSS and the two adjacent bins; "5 bins average" is the average of the 100 kb bin containing the gene's TSS and the adjacent four bins.

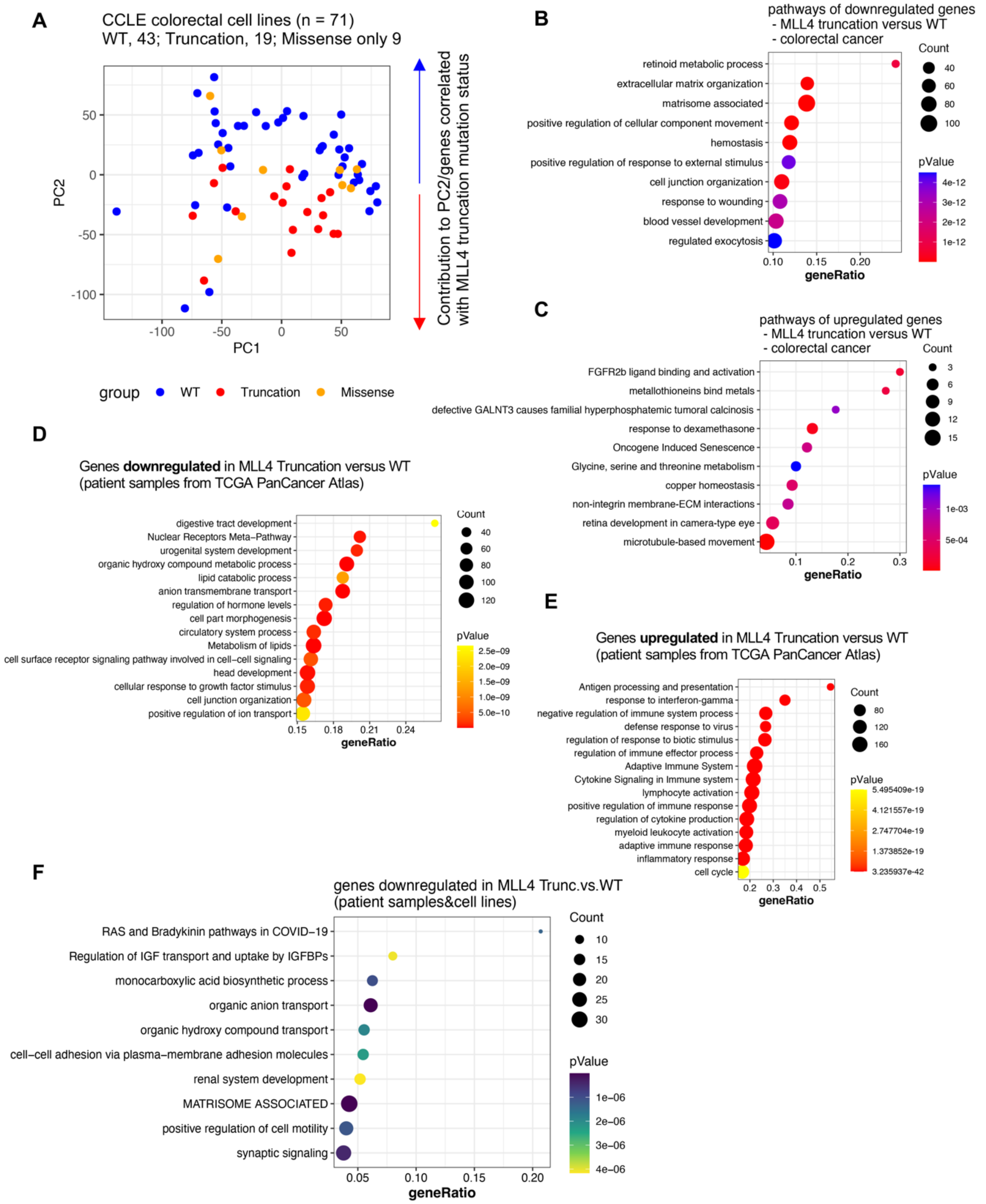
A
CCLE colorectal cell lines (n = 71)
WT, 43; Truncation, 19; Missense only 9
PC1
PC2
Contribution to PC2/genes correlated with MLL4 truncation mutation status
group WT Truncation Missense
B
pathways of downregulated genes
- MLL4 truncation versus WT
- colorectal cancer
retinoid metabolic process
extracellular matrix organization
matrisome associated
positive regulation of cellular component movement
hemostasis
positive regulation of response to external stimulus
cell junction organization
response to wounding
blood vessel development
regulated exocytosis
geneRatio
Count
pValue
C
pathways of upregulated genes
- MLL4 truncation versus WT
- colorectal cancer
FGFR2b ligand binding and activation
metallothioneins bind metals
defective GALNT3 causes familial hyperphosphatemic tumoral calcinosis
response to dexamethasone
Oncogene Induced Senescence
Glycine, serine and threonine metabolism
copper homeostasis
non-integrin membrane-ECM interactions
retina development in camera-type eye
microtubule-based movement
geneRatio
Count
pValue
D
Genes **downregulated** in MLL4 Truncation versus WT
(patient samples from TCGA PanCancer Atlas)
digestive tract development
Nuclear Receptors Meta-Pathway
urogenital system development
organic hydroxy compound metabolic process
lipid catabolic process
anion transmembrane transport
regulation of hormone levels
cell part morphogenesis
circulatory system process
Metabolism of lipids
cell surface receptor signaling pathway involved in cell-cell signaling
head development
cellular response to growth factor stimulus
cell junction organization
positive regulation of ion transport
geneRatio
Count
pValue
E
Genes **upregulated** in MLL4 Truncation versus WT
(patient samples from TCGA PanCancer Atlas)
Antigen processing and presentation
response to interferon-gamma
negative regulation of immune system process
defense response to virus
regulation of response to biotic stimulus
regulation of immune effector process
Adaptive Immune System
Cytokine Signaling in Immune system
lymphocyte activation
positive regulation of immune response
regulation of cytokine production
myeloid leukocyte activation
adaptive immune response
inflammatory response
cell cycle
geneRatio
Count
pValue
F
genes downregulated in MLL4 Trunc.vs.WT
(patient samples&cell lines)
RAS and Bradykinin pathways in COVID-19
Regulation of IGF transport and uptake by IGFBPs
monocarboxylic acid biosynthetic process
organic anion transport
organic hydroxy compound transport
cell-cell adhesion via plasma-membrane adhesion molecules
renal system development
MATRISOME ASSOCIATED
positive regulation of cell motility
synaptic signaling
geneRatio
Count
pValue

Supplemental Figure 10. Loss-of-function MLL4 mutations in colorectal cancer share similar gene expression features with mESC depleted of MLL3/4. (**A**) PCA of 71 colorectal cancer cell lines in the Cancer Cell Line Encyclopedia (CCLE) based on RNA-seq gene-expression data obtained from DepMap. Cell lines were grouped based on the MLL4 mutation status: WT, n= 43; Truncation, n = 19; Missense only, n = 9. If truncation mutation and missense mutation co-exist in one cell line, it is annotated as truncation. PC1, 24% variance; PC2, 11% variance. (**B**) Pathway analysis of genes downregulated in MLL4 truncation versus WT CCLE colorectal cancer cell lines. (**C**) Pathway analysis of genes upregulated in MLL4 truncation versus WT CCLE colorectal cancer cell lines. (**D**) Pathway analysis of genes downregulated in MLL4 truncation versus WT patient samples from the TCGA PanCancer Atlas. (**E**) Pathway analysis of genes upregulated in MLL4 truncation versus WT patient samples from the TCGA PanCancer Atlas. (**F**) Pathway analysis of genes downregulated in the MLL4 truncation versus WT condition in both CCLE colorectal cancer cell lines and patient samples from the TCGA PanCancer Atlas. Data were retrieved from cBioPortal with OQL (Onco Query language) to define MLL4 mutation status (https://www.cbioportal.org/).

A

Cell Line	FHC, normal	Caco2	SW1417	HT55	SW480	DLD1	HCT116	RKO
**KMT2D**	-	WT	WT	WT	D1633N	F5284L, L2304M, P1931H, E1667D, E1517D	R2443Sfs*6, V160M	L5056P, G3465*, P2550Lfs*33, L1600P, R1189H
**KMT2C**	-	S2427G	S1416L, P2050L	G1584L	Q4279*	G3438D, I1706V	I1344*	R3853W
**KDM6A**	-	WT	WT	WT	WT	WT	WT	WT

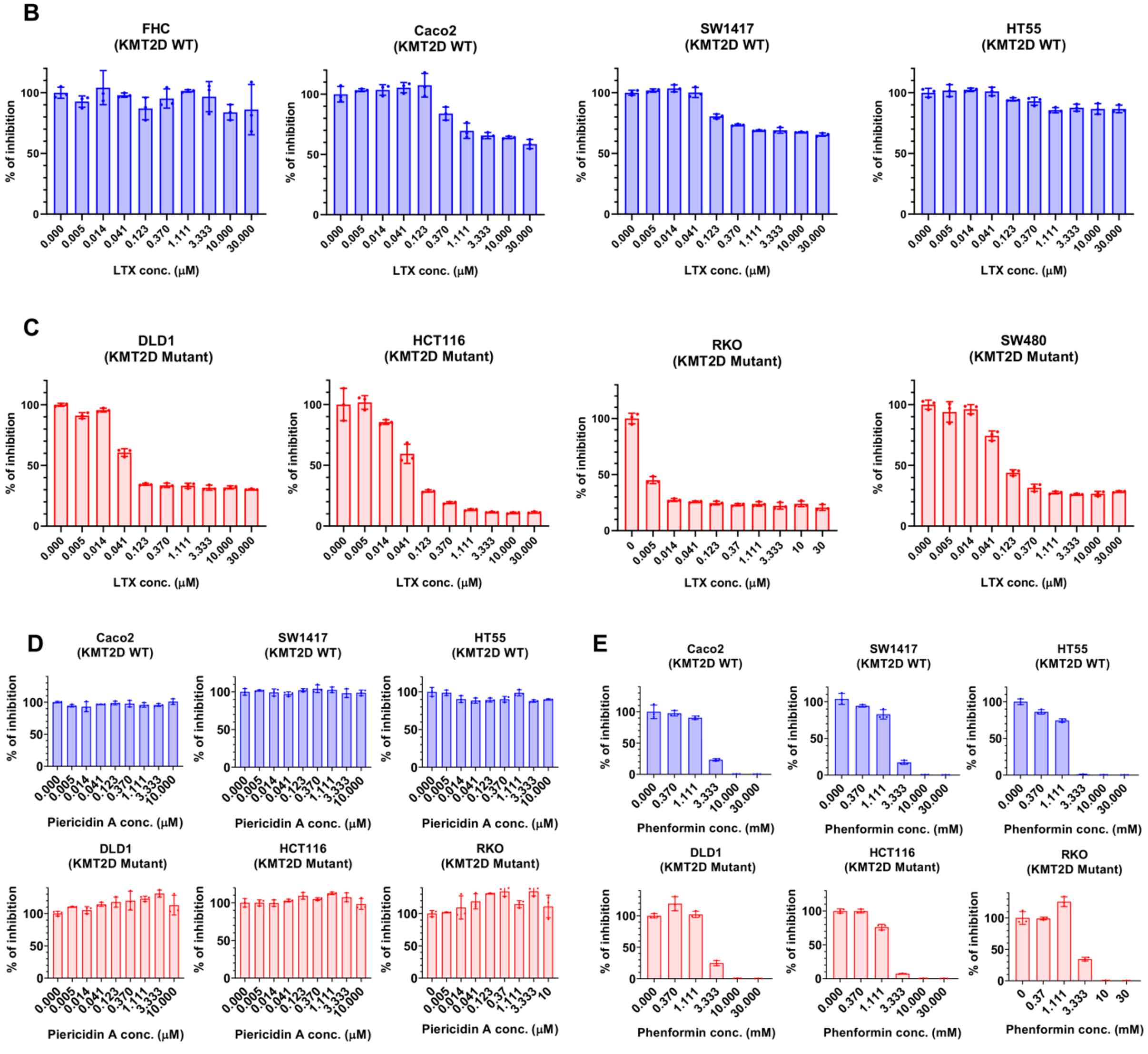

Supplemental Figure 11. MLL4 mutant colorectal cancer cells are selectively sensitive to lometrexol treatment. (**A**) MLL3/4 and UTX mutation status of the colorectal cell lines. (**B**-**C**) Cell growth inhibition upon LTX treatment (0 – 30 μM) in MLL4 wild type (**B**) and MLL4 mutant (**C**) cell lines. (**D-E**) Cell growth inhibition upon treatment with Piericidin A (0 – 10 μM) **(D)** or Phenformin (0 – 30 mM) (**E**) in MLL4 wild type and MLL4 mutant cell lines. CellTiter-Glo® luminescent cell viability assays were performed 72 hours after treatment to determine the percentage of inhibition under each treatment condition. Data are presented as mean ± standard deviation (SD) with n=3. *$P < 0.05$, **$P < 0.01$, ***$P < 0.001$ with unpaired t-test.

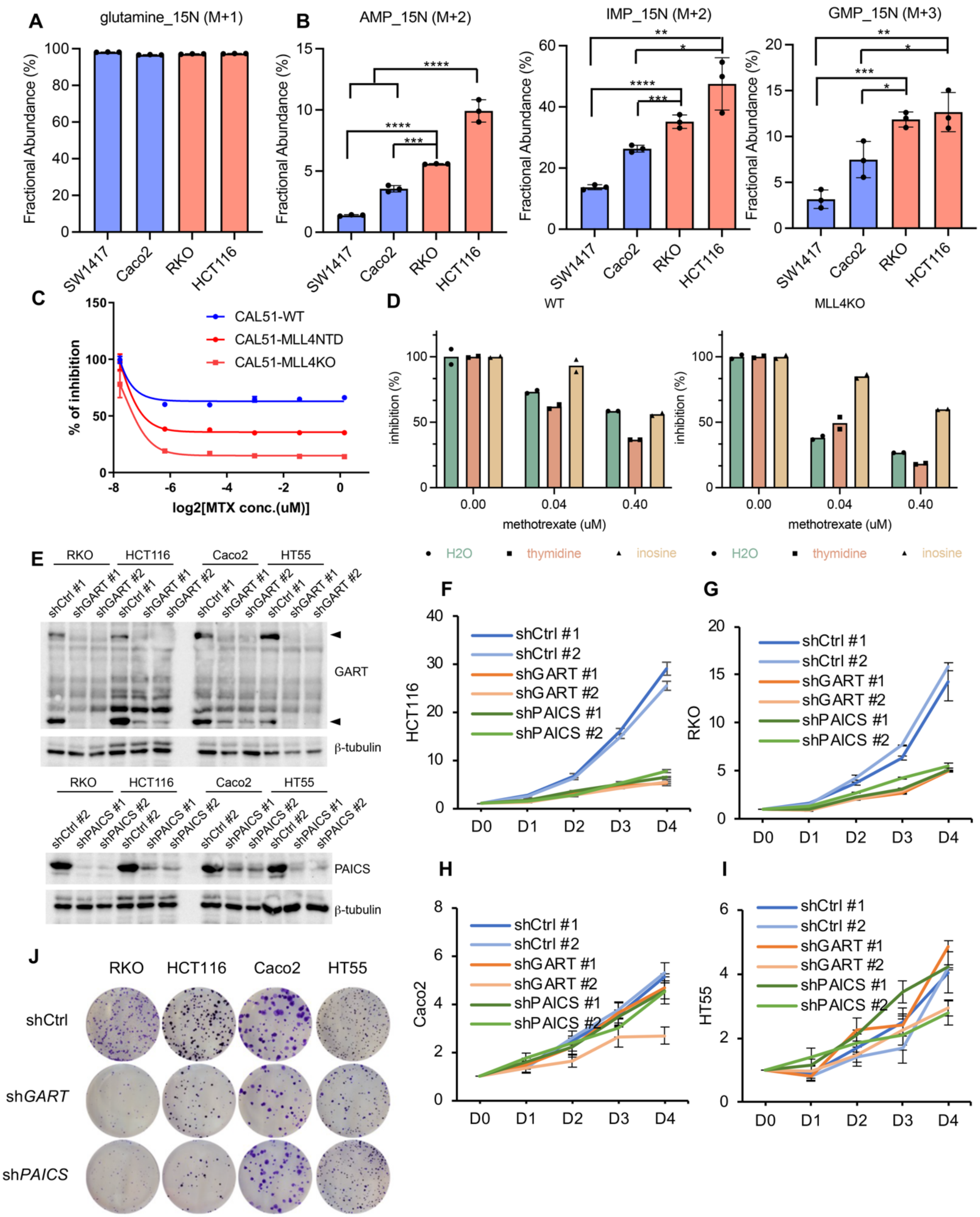
A
glutamine_15N (M+1)
Fractional Abundance (%)
SW1417
Caco2
RKO
HCT116
B
AMP_15N (M+2)
IMP_15N (M+2)
GMP_15N (M+3)
C
CAL51-WT
CAL51-MLL4NTD
CAL51-MLL4KO
% of inhibition
log2[MTX conc.(uM)]
D
WT
MLL4KO
inhibition (%)
methotrexate (uM)
H2O
thymidine
inosine
E
GART
β-tubulin
PAICS
F
G
H
I
shCtrl #1
shCtrl #2
shGART #1
shGART #2
shPAICS #1
shPAICS #2
HCT116
RKO
Caco2
HT55
D0
D1
D2
D3
D4
J
shCtrl
shGART
shPAICS

Supplemental Figure 12. Cells with compromised MLL4 function are selectively sensitive to de novo purine synthesis inhibition. (**A**) Tracer levels of glutamine (M+1) in SW1417, Caco2, RKO and HCT116 cells. (**B**) Incorporation of ^{15}N tracer from glutamine into purines in SW1417, Caco2, RKO and HCT116 cells. MLL4 WT cells, blue; MLL4 mutant cells, red. Data are presented as mean ± standard deviation (SD) with n=3. *P < 0.05, **P < 0.01, ***P < 0.001, ****P < 0.0001 with unpaired t-test. (**C**) A CellTiter-Glo® luminescent cell viability assay was performed in CAL51 WT, heterozygous MLL4 truncation mutation (hNTD), and MLL4 KO cells treated with (MTX) (0 – 1 µM). n=3. (**D**) A CellTiter-Glo® luminescent cell viability assay was performed in CAL51 WT and MLL4 KO cells were treated with 0, 0.04, or 0.4 µM MTX in the presence of H2O, 50 µM thymidine or 50 µM inosine. n=2. (**E-J**) Cells were infected with lentiviruses expressing shCtrl, shGART or shPAICS. Two distinct shRNAs were included for both GART and PAICS. Western blot showing the knockdown of GART and PAICS in RKO, HCT116, Caco2 and HT55 cells (**E**). The growth rates of HCT116 (**F**), RKO (**G**), Caco2 (**H**) and HT55 (**I**) cells with shCtrl, shGART or shPAICS were measured by cell counting (n=2), and the cell number was normalized to day 0. Colony formation in the four cell lines with GART or PAICS knockdown was performed by seeding 500 or 1000 cells in 6 well plates and staining after 10-14 days of culture with media changed every three days (**J**).

A

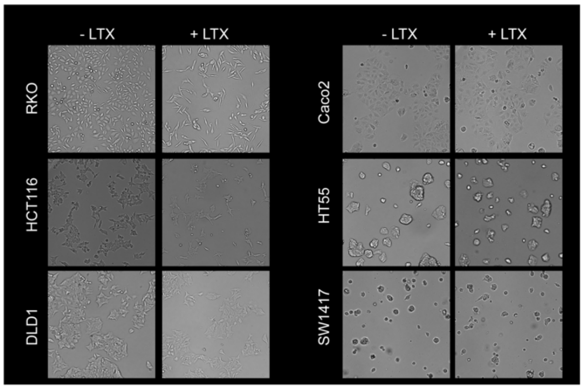

B

Line	KMT2D	direction	LTX_vs_DMSO
RKO	Mut	up	2922
RKO	Mut	dn	2980
HCT116	Mut	up	3642
HCT116	Mut	dn	3528
DLD1	Mut	up	2147
DLD1	Mut	dn	2096
HT55	WT	up	1
HT55	WT	dn	0
Caco2	WT	up	22
Caco2	WT	dn	3
SW1417	WT	up	91
SW1417	WT	dn	53

C

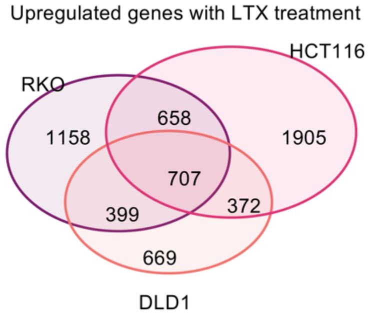

D

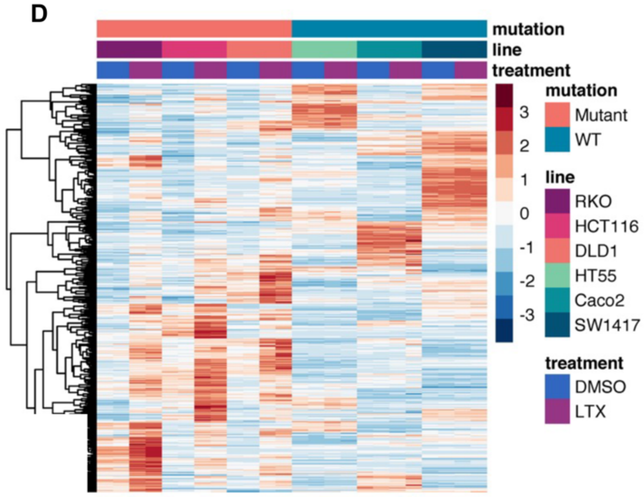

Commonly upregulated gene expression in LTX_vs_DMSO in RKO, HCT116, and DLD1 (n = 707)

E

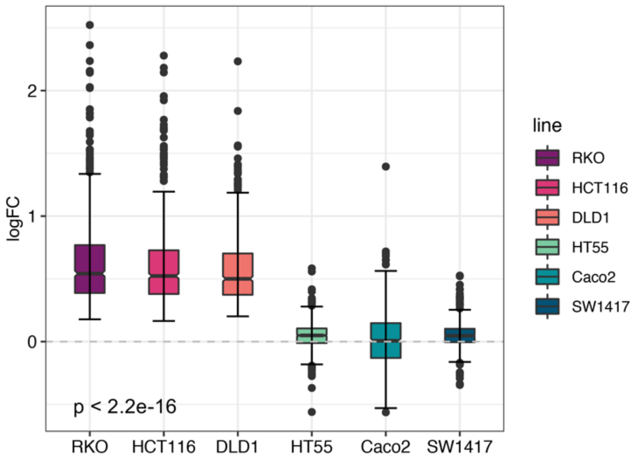

Supplemental Figure 13. RNA-seq data in colorectal cancer cells treated with LTX. (**A**) Morphological change in colorectal cancer cell lines treated with LTX at 1μM for 24 hours viewed under bright field microscope. (**B**) summary of total differentially expressed gene numbers (up and down-regulated genes separated) in these cell lines, with MLL4 mutation status indicated. adj.p < 0.01. (**C**) Venn diagram showing the overlap of upregulated genes in LTX treated versus control in RKO, HCT116, and DLD1. (**D**) Hierarchical clustering heatmap showing the expression of genes upregulated in LTX treated versus control in common among RKO, HCT116 and DLD1 cell lines (n = 707). (**E**) Box plot showing the logFC of the 707 genes upregulated in common among RKO, HCT116 and DLD1 cells upon LTX treatment. An unpaired t-test was used to calculate the p value. Gene expression was significantly different compared with MLL4 WT cells for all the MLL4 mutant cell lines.

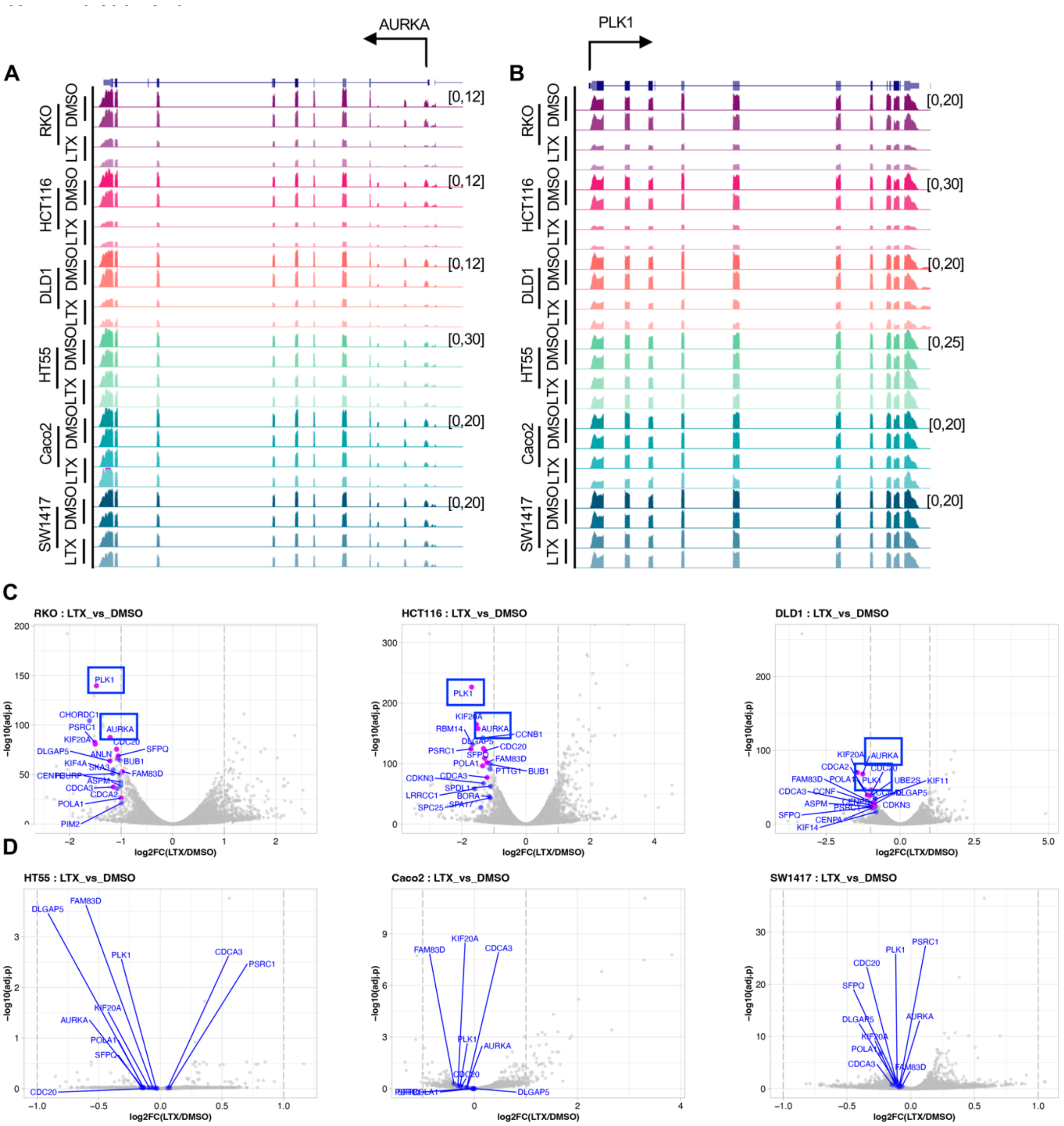
A
AURKA
B
PLK1
RKO
HCT116
DLD1
HT55
Caco2
SW1417
DMSO
LTX
[0,12]
[0,30]
[0,20]
[0,25]
C
RKO : LTX_vs_DMSO
HCT116 : LTX_vs_DMSO
DLD1 : LTX_vs_DMSO
D
HT55 : LTX_vs_DMSO
Caco2 : LTX_vs_DMSO
SW1417 : LTX_vs_DMSO
-log10(adj.p)
log2FC(LTX/DMSO)
PLK1
AURKA

Supplemental Figure 14. The unique gene expression signature of MLL4 mutant cells in response to LTX treatment. (**A-B**) RNA-seq track examples showing the AURKA (**A**) and PLK1 (**B**) gene expression in MLL 4 mutant (DLD1, HCT116, RKO) or WT (SW1417, Caco2, HT55) colorectal cancer cell lines in response to LTX treatment. (**C**) A collection of 217 genes related to mitotic cell cycle pathways was selected for visualization. Volcano plots highlighting the top 20 downregulated genes involved in mitotic cell cycle pathways in MLL4 mutant cell lines (RKO, HCT116 and DLD1). The 10 genes found in common among the plots (PLK1, AURKA, CDCA3, CDC20, SFPQ, POLA1, PSRC1, KIF20A, FAM83D, and DLGAP5) were defined as the "lometrexol responsive mitotic gene signature" (**D**) Volcano plots showing the expression of these 10 genes in MLL4 WT cell lines (HT55, Caco2 and SW1417).

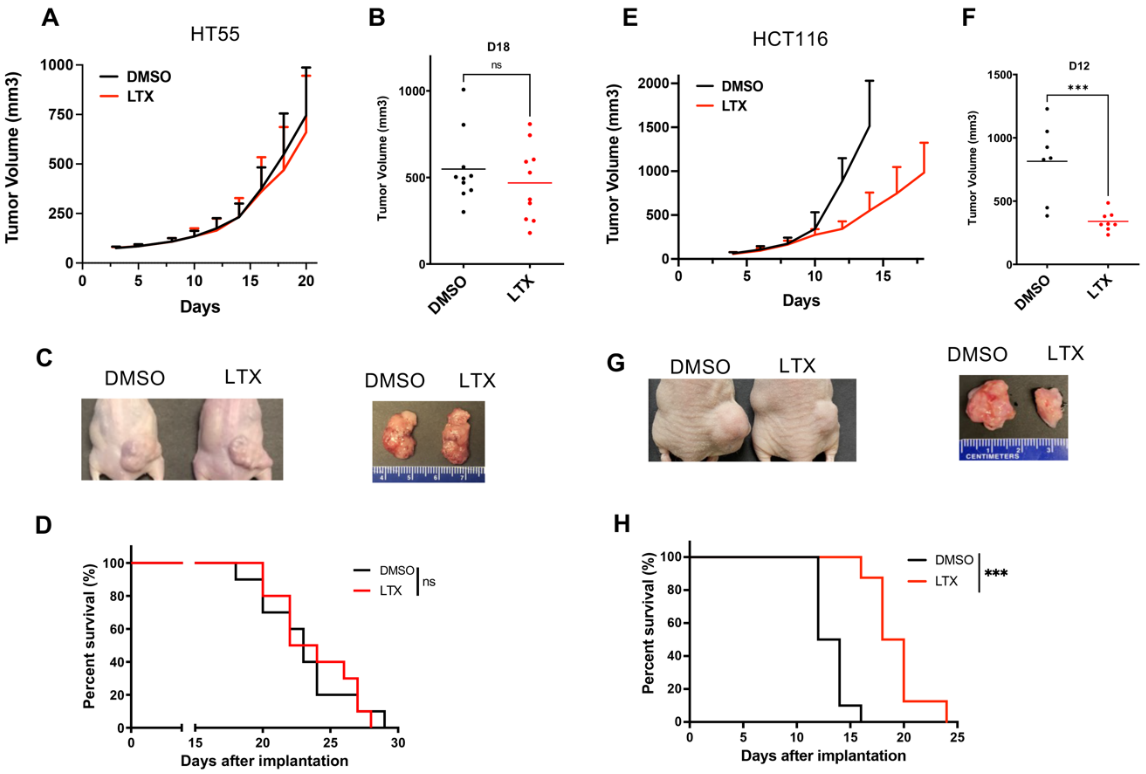
A
HT55
Tumor Volume (mm3)
Days
DMSO
LTX
B
D18
ns
Tumor Volume (mm3)
DMSO
LTX
E
HCT116
Tumor Volume (mm3)
Days
F
D12
***
C
DMSO
LTX
G
CENTIMETERS
D
Percent survival (%)
Days after implantation
H
***

Supplemental Figure 15. Lometrexol application in MLL4 mutant tumors in the xenograft mouse models. (**A-D**) Tumor development after inoculation of 4×10^6 of HT55 cells into nude mice. Mice with HT55 subcutaneous (sc) tumors were treated with either vehicle (DMSO, n = 9) or LTX (15 mg/kg, n = 9) daily for 7 days. (**A**) Growth plots for sc tumors in each treatment group are shown with mean tumor volumes (mm^3 and upper SD). (**B**) Dot plot representation of sc tumor volume in mice at day 18 post-tumor cell injection. Unpaired t-test values for comparisons between DMSO and LTX treatment: ns, not statistically significant. (**C**) Photographs of nude mice (left) in which HT55 cells were inoculated into the right flank and sc tumors taken from these mice (right). (**D**) Animal survival at the indicated days after inoculation. Log-rank test was used for comparisons between DMSO and LTX treatment. (**E-H**) Tumor development after inoculation of 4×10^6 of HCT116 cells into nude mice. Mice with HCT116 subcutaneous (sc) tumors were treated with either vehicle (DMSO, n = 9) or LTX (15 mg/kg, n = 9) daily for 7 days. (**E**) Growth plots for sc tumors in each treatment group are shown with mean tumor volumes (mm3) and upper SD. (**F**) Dot plot representation of sc tumor volume in mice at day 12 post-tumor cell injection. Unpaired t-test values for comparisons between DMSO and LTX treatment: ***, $P < 0.001$. (**G**) Photographs of nude mice (left) in which HCT116 cells were inoculated into the right flank and sc tumors taken from these mice (right). (**H**) Animal survival at the indicated days after inoculation. Log-rank test was used for comparisons between DMSO and LTX treatment: ***, $P < 0.001$.

Table S1.

Mouse shRNA					
Clone ID	Oligo Seq	Symbol	RefSeq ID	Gene ID	Gene Description
TRCN0000081503	CCGGGCAGGAACAGAAGTTCGCTATCTCGAGATAGCGAACTTCTGTTCCTGCTTTTTG	Alpl	NM_007431	11647	alkaline phosphatase 2, liver
TRCN0000080349	CCGGCCTAGACAACGACAAGTACATCTCGAGATGTACTTGTCGTTGTCTAGGTTTTTG	Sparc	NM_009242	20692	secreted acidic cysteine rich glycoprotein
TRCN0000081712	CCGGCTTCGACCAAACCGTTTCTCTCTCGAGAGAGAAACGGTTTGGTCGAAGTTTTTG	Utf1	NM_009482	22286	undifferentiated embryonic cell transcription factor 1
TRCN0000102500	CCGGGCCTTGTTGATCTATCTTGTACTCGAGTACAAGATAGATCAACAAGGCTTTTTG	Dazl	NM_010021	13164	deleted in azoospermia-like
TRCN0000105190	CCGGGCCTACTACCATCATCGAGAACTCGAGTTCTCGATGATGGTAGTAGGCTTTTTG	Fabp3	NM_010174	14077	fatty acid binding protein 3, muscle and heart
TRCN0000340859	CCGGAGCAAGCACTAAGGATCAAAGCTCGAGCTTTGATCCTTAGTGCTTGCTTTTTTG	Map6	NM_010837	17760	microtubule-associated protein 6
TRCN0000331554	CCGGGATTCGAGAGACAGAGGTCATCTCGAGATGACCTCTGTCTCTCGAATCTTTTTG	Sdc4	NM_011521	20971	syndecan 4
TRCN0000097583	CCGGCGCTTAAATGCCGGAGAAGTTCTCGAGAACTTCTCCGGCATTTAAGCGTTTTTG	Bhmt	NM_016668	12116	betaine-homocysteine methyltransferase
TRCN0000108475	CCGGGCCTTTAATATGGACCCTGTTCTCGAGAACAGGGTCCATATTAAAGGCTTTTTG	Ddc	NM_016672	13195	dopa decarboxylase
TRCN0000039105	CCGGCTTCTGGATATTCATGGACAACTCGAGTTGTCCATGAATATCCAGAAGTTTTTG	Dnmt3l	NM_019448	54427	DNA (cytosine-5-)-methyltransferase 3-like
TRCN0000173430	CCGGCCTAGAGTACAGGGTGAACATCTCGAGATGTTCACCCTGTACTCTAGGTTTTTTG	Ncoa6	NM_019825	56406	nuclear receptor coactivator 6
TRCN0000446257	CCGGGGCGAGCTGAGATTTGGATATCTCGAGATATCCAAATCTCAGCTCGCCTTTTTG	Khdc3	NM_025890	66991	RIKEN cDNA 2410004A20 gene
TRCN0000200876	CCGGGAAGAGAAGTCTTCGTCAATTCTCGAGAATTGACGAAGACTTCTCTTCTTTTTG	Hormad1	NM_026489	67981	HORMA domain containing 1
TRCN0000110910	CCGGCGCTAATTTGATCCTGTGTTTCTCGAGAAACACAGGATCAAATTAGCGTTTTTG	Glipr2	NM_027450	384009	GLI pathogenesis-related 2
TRCN0000126003	CCGGCGAGACCCATTGGCAATACTACTCGAGTAGTATTGCCAATGGGTCTCGTTTTTG	Susd2	NM_027890	71733	sushi domain containing 2
TRCN0000112644	CCGGCGGGACGCCTTCGATACTCTTCTCGAGAAGAGTATCGAAGGCGTCCCGTTTTTG	Parvb	NM_133167	170736	parvin, beta

TRCN0000042657	CCGGGCTGACATACATTGATGACAACTCGAGTTGTCATCAATGTATGTCAGCTTTTTG	Mcf2	NM_133197	109904	mcf.2 transforming sequence
TRCN0000420527	CCGGCACCTTCATGTCTGCATATTTCTCGAGAAATATGCAGACATGAAGGTGTTTTTG	Cth	NM_145953	107869	cystathionase (cystathionine gamma-lyase)
TRCN0000080513	CCGGCCAGTGATGCTGTAAGTCATTCTCGAGAATGACTTACAGCATCACTGGTTTTTG	Cd109	NM_153098	235505	CD109 antigen
Human shRNA					
Clone ID	Target Sequence	Symbol	RefSeq ID	Gene ID	Gene Description
TRCN0000289428	CCCTAACTGTTGTCATGGCAA	GART #1	NM_000819	2618	AIRS, GARS, GARTF, PAIS, PGFT, PRGS
TRCN0000289431	GCACAGTCTCATCATGTCAAA	GART #2	NM_000819	2618	AIRS, GARS, GARTF, PAIS, PGFT, PRGS
TRCN0000045774	CGCAGTGTGAAATGATTCCAA	PAICS #1	NM_006452	10606	ADE2, ADE2H1, AIRC, PAIS
TRCN0000045775	GCTGCTCAGATATTTGGGTTA	PAICS #2	NM_006452	10606	ADE2, ADE2H1, AIRC, PAIS
qRT-PCR primers					
Ak3-QF	GCCTGAAGGGATGTGGTATTAG				
Ak3-QR	CCTGTTAAGGTAGCAGTGAGTT				
Nqo1-QF	GAGAAGAGCCCTGATTGTACTG				
Nqo1-QR	ACCTCCCATCCTCTCTTCTT				
Gapdh-QF	AACAGCAACTCCCACTCTTC				
Gapdh-QR	CCTGTTGCTGTAGCCGTATT				

References

1. K. Cao *et al.*, An MLL4/COMPASS-Lsd1 epigenetic axis governs enhancer function and pluripotency transition in embryonic stem cells. *Sci Adv* **4**, eaap8747 (2018).
2. G. Yu, L. G. Wang, Q. Y. He, ChIPseeker: an R/Bioconductor package for ChIP peak annotation, comparison and visualization. *Bioinformatics* **31**, 2382-2383 (2015).
3. R. Rickels *et al.*, Histone H3K4 monomethylation catalyzed by Trr and mammalian COMPASS-like proteins at enhancers is dispensable for development and viability. *Nat Genet* **49**, 1647-1653 (2017).
4. J. H. Gibcus *et al.*, A pathway for mitotic chromosome formation. *Science* **359**, (2018).
